# A combination of long term fragmentation and glacial persistence drove the evolutionary history of the Italian wall lizard *Podarcis siculus*

**DOI:** 10.1186/s12862-016-0847-1

**Published:** 2017-01-05

**Authors:** Gabriele Senczuk, Paolo Colangelo, Emanuela De Simone, Gaetano Aloise, Riccardo Castiglia

**Affiliations:** 1 Dipartimento di Biologia e Biotecnologie “Charles Darwin”, Università di Roma LA SAPIENZA, sede di Anatomia comparata, Rome, Italy; 2 National Research Council, Institute of Ecosystem Study, Largo Tonnoli 50, 28922 Verbania Pallanza, Italy; 3 Museo di Storia Naturale della Calabria e Orto Botanico, Università della Calabria, CAP 87036 Rende, Cosenza Italy

**Keywords:** Italian wall lizard, Phylogeography, Glacial expansion, *Podarcis siculus*, Niche modelling, Evolutionary history

## Abstract

**Background:**

The current distribution of genetic diversity is the result of a vast array of microevolutionary processes, including short-term demographic and ecological mechanisms and long-term allopatric isolation in response to Quaternary climatic fluctuations. We investigated past processes that drove the population differentiation and spatial genetic distribution of the Italian wall lizard *Podarcis siculus* by means of sequences of mitochondrial *cytb* (*n* = 277 from 115 localities) and nuclear *mc1r* and *β-fibint7*genes (*n* = 262 and *n* = 91, respectively) from all its distribution range. The pattern emerging from the genetic data was compared with current and past (last glacial maximum) species distribution modeling (SDM).

**Results:**

We identified seven deeply divergent parapatric clades which presumably remained isolated in different refugia scattered mainly throughout the Tyrrhenian coast. Conversely, the Adriatic coast showed only two haplogroups with low genetic variability. These results appear to agree with the SDM prediction at the last glacial maximum (LGM) indicating a narrow area of habitat suitability along the Tyrrhenian coast and much lower suitability along the Adriatic one. However, the considerable land exposure of the Adriatic coastline favored a glacial colonization of the Balkan Peninsula.

**Conclusions:**

Our population-level historical demography showed a common trend consistent with glacial expansions and regional persistence during the last glacial maximum. This complex genetic signature appears to be inconsistent with the expectation of the expansion-contraction model and post-LGM (re)colonizations from southern refugia. Hence it is one of an increasing number of cases in which these assumptions are not met, indicating that long-term fragmentation and pre-LGM events such as glacial persistence were more prominent in shaping genetic variation in this temperate species.

**Electronic supplementary material:**

The online version of this article (doi:10.1186/s12862-016-0847-1) contains supplementary material, which is available to authorized users.

## Background

The cyclic population expansions and contractions primed by the Quaternary climatic oscillation are considered one of the most important processes that led to the current distribution of genetic variation across different geographic areas [1–5]. However, the huge amount of molecular data made available in recent years has led to a vast array of reconsiderations of microevolutionary processes that underlie the Pleistocene biogeography. Species responses to past climatic changes have been shown to be more variable than formerly thought, depending on species-specific physiological and ecological requirements [6–10]. Moreover, the climatic fluctuations acting in different and restricted topographic contexts generated a variety of contrasting evolutionary histories even at the population level [11, 12].

In the Mediterranean region, evidence from species inhabiting temperate and coastal areas suggested a certain flexibility of the expansion-contraction (EC) model of Pleistocene biogeography [1] and of the expectation of post-last glacial maximum (LGM) demographic expansions from southern refugia. These new perspectives suggest that some temperate species could have undergone attenuated or even reverse responses to glacial population contractions, with the absence of a post-LGM (re)colonization pattern [13, 14]. This scenario appears plausible considering the strong sea level decrease (as much as 120–135 m) during the LGM, leading to the increase of Mediterranean coastal plains and favoring the formation of new suitable habitats with consequent population expansions.

Moreover, climate-driven cycles of allopatric differentiation within sub-refugia have now become the rule rather than the exception in molding patterns of genetic diversity in many Mediterranean species. Meanwhile, the perception of the maintenance of genetic diversity within a single macrorefugium, induced by population demographic stability, has gradually diminished in favor of the alternative ‘refugia within refugia’ scenario [15]. In fact, a growing number of taxa reveal evidence of strong phylogeographic structure within the main southern regions ([16–18] in the Iberian Peninsula; [19, 20] in the Italian Peninsula; [11, 21, 22] in the Balkan Peninsula).

The Italian Peninsula is considered an important refugial area for many Mediterranean species due to its complex topography and wide latitudinal range [7, 23]. It has been repeatedly used as a study area to gain new insights into alternative microevolutionary processes generating patterns of genetic diversity [24]. Most of these studies confirmed a southern richness pattern of genetic diversity. In particular, the southernmost portion of the Italian Peninsula, the “Calabrian arc”, has been called a melting pot of intraspecific diversity [25]. From the Pliocene to the Middle Pleistocene, especially during high-sea level interglacials, this area consisted of different islands corresponding to the Aspromonte, Sila and Pollino massifs [26, 27]. The rise of highly divergent genetic lineages in these areas is probably closely linked to these paleo-island systems. However, the northern and central Apennines have also played a role in generating long-term genetic discontinuity, suggesting the presence of additional cryptic refugia in the central and northern portions of the Italian Peninsula [28, 29].

On the whole, the genetic population structure of many species inhabiting the Italian Peninsula suggests a vast assortment of long-term allopatric fragmentations and recent short-term distributional and demographic rearrangements in response to environmental variability.

In the present study, we used the Italian wall lizard *Podarcis siculus* to investigate a part of this microevolutionary process by means of mtDNA and nuDNA sequences in a phylogeographic framework. The Italian wall lizard is a good model to elucidate the historical events shaping the genetic architecture in the Italian Peninsula. It is widespread and very abundant in peninsular Italy, on the large islands Sicily, Sardinia and Corsica, and along the northern part of the eastern Adriatic coast and many Adriatic islands. Podnar et al. [30] recognized six main clades on the basis of mitochondrial data: three in southern Italy (Sicily and Calabria) and three in central-northern Italy and Croatia. Although their study included many Adriatic regions of Croatia and some southern Italian populations, much of the Italian peninsular area was not investigated.

Therefore, the aim of our study was to determine the long-term or recent microevolutionary processes most important in shaping the current genetic architecture across the entire refugial area. In doing so we wished to (i) assess the different demographic trends, distinguishing long-term isolation and/or allopatric differentiation within sub-refugia from recent expansion events, and (ii) shed more light on the importance of the Pleistocene environmental changes and consequent microevolutionary processes in structuring biodiversity in the Italian Peninsula.

## Methods

### Sampling, laboratory procedures and molecular data

**Fig. 1:**
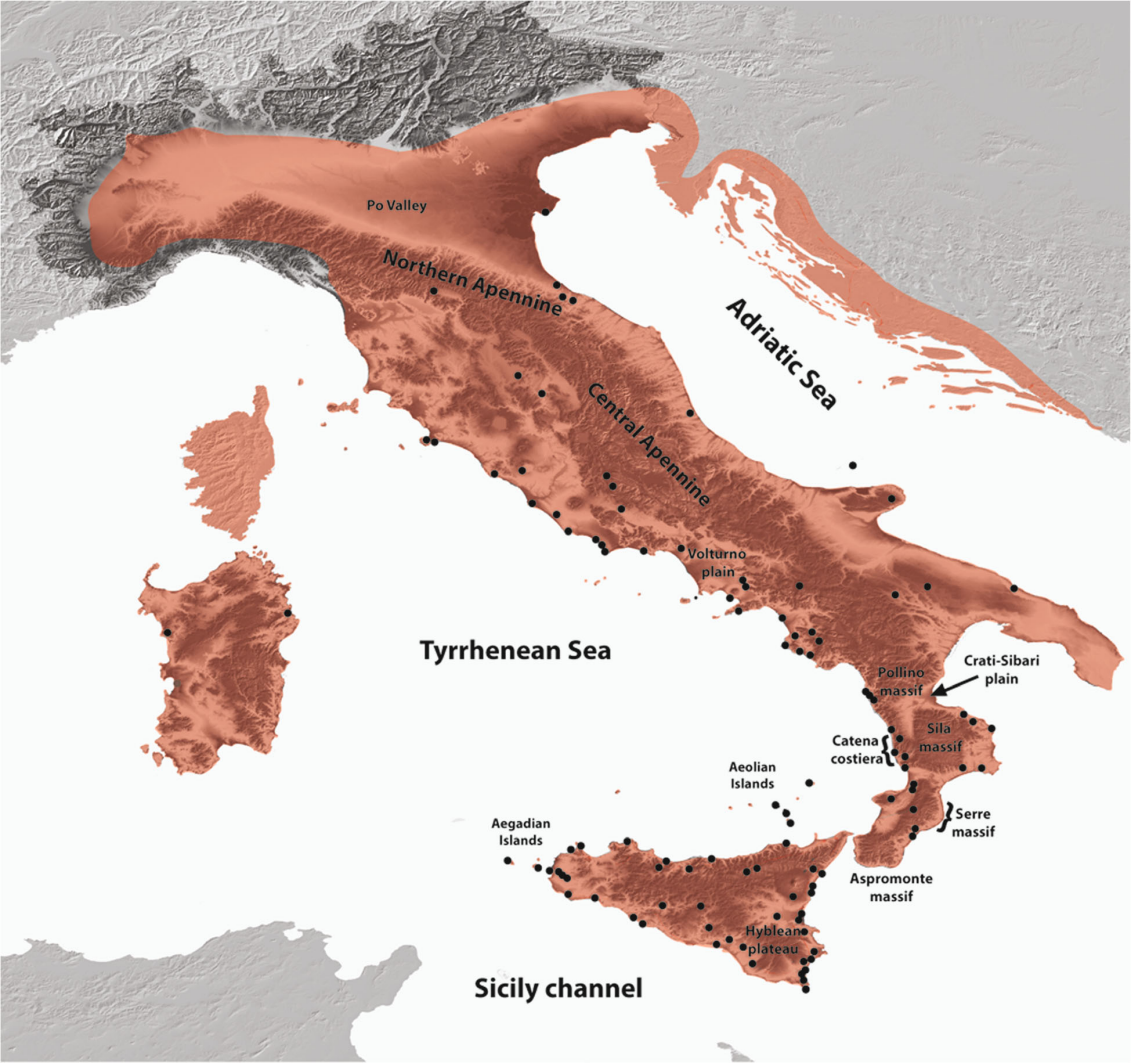
Map of the study area showing the principal geographic features mentioned in the main text. The area in *red* corresponds to the geographic distribution of *Podarcis siculus* while the 115 new sampled locations are shown in *black dots*

The sampling took place from March 2013 to June 2015, during which we collected 277 samples from 115 localities covering most of the distribution area of *Podarcis siculus* in the Italian Peninsula and Sicily. The tissues were obtained by inducing autotomy after light pressure and were then stored in 96% pure ethanol. All lizards were released at the capture location. The sampling was planned to complement already available data in order to achieve a better geographic coverage of the species distribution [30]. The geographic coordinates were recorded and high-resolution photos were taken for each individual. The geographic references and sample size of all sampled populations are given in Additional file 1: Table S1 and shown in Fig. 1.

Genomic DNA was extracted from all the tissue samples by means of the universal extraction protocol of Salah et al. [31] with incubation at 56 °C with proteinase K and DNA precipitation with isopropanol. The total genome was visually checked in electrophoretic runs. We used standard PCR protocols to amplify the mitochondrial fragment of cytochrome b (*cytb*) and two nuclear fragments, the Melanocortin receptor 1 (*mc1r*) and the β-fibrinogen intron 7 (*β-fibint7*). The primers used for the amplifications and their respective references are reported in Table 1. As the presence of a pseudogene for *cytb* has been reported in this species [32], we modified the primers to be strongly specific to amplify only the mitochondrial *cytb* sequences.

**Table 1:** Pairs of primers used in this study with relative references

Gene	Primer	Primer sequence	Reference
*cytb*	CytF	5′ - TTTGGATCACTATTRGGCCTCTGCC - 5′	modified in this study from [30]
	H15425	5′- GGTTTACAAGACCAGTGCTTT-3′	
*mc1r*	MC1R-PF	5′- GGCNGCCATYGTCAANAACCGGAACC - 3′	[103]
	MC1R-PR	5′- CTCCGRAAGGCRTAAATNATGGGGTCCAC - 3′	
*β-fibint7*	BFXF	5′ - CAGYACTTTYGAYAGAGACAAYGATGG - 3′	[104]
	FIB B17L	5′ - TCCCCAGTAGTATCTGCCATTAGGGTT - 3′	[105]

The resulting PCR products were purified with a Sure Clean (Bioline) purification kit. Sequencing reactions were run under Big-Dye TM Terminator cycling conditions by the commercial company Macrogen (www.macrogen.com). The electropherograms were then checked using FinchTV and minor changes were made by eye. IUPAC ambiguity codes were used to represent polymorphism of heterozygote individuals in nuclear sequences. For the nuclear sequences, we solved the gametic phases using the coalescent-based Bayesian algorithm in PHASE v.2.1 [33, 34] as implemented in DnaSPv.5.1 [35]. Since the *β-fibint7* showed the presence of INDELs polymorphisms, we first used the method described by Flot et al. [36] to determine the phase for sequences that were heterozygous for insertion or deletions (12 individuals). We then implemented the known phases in the coalescent-based Bayesian reconstruction. Three independent runs were conducted with burn-in at 1000 and 10,000 iterations, and thinning at each 100 steps. Only sequences with posterior probability >0.75 were included in the analysis. A final consensus alignment was computed for each marker with MEGA 5.0 [37]. When the final alignments were obtained, the number of haplotypes (H), nucleotides (π) and haplotype (h) diversity were estimated using DnaSPv.5.1 for the overall mtDNA and nuDNA datasets and for each mtDNA clade revealed by the phylogenetic analysis. Since the statistical power of tests for recombination is generally low, two different methods were used to assess for nuclear recombination. The four-gamete test was performed to estimate the minimum number of recombination events obtaining confidence intervals at 95% by the coalescent algorithm implemented in DnaSP v.5.1.

Moreover, we also test the occurrence of recombination events through the Pairwise Homoplasy Index (phi) test implemented in the program splitstree v. 4 [38].

### Phylogeographic structureand time of divergence

The phylogenetic analyses were carried out using a data set including *cytb* sequences generated in this study (*n* = 277 specimens) and those available in GenBank (*n* = 76 specimens) from Podnar et al. [30], for a total alignment of 353 sequences. For the nuclear markers, here generated, we used a total of 524 sequences for *mc1r* and 182 sequences for the *β-fibint7* (accession numbers for each gene are reported in Additional file 2: Table S2).

To evaluate the best fitting substitution model for *cytb*, we used jModeltestv.2 [39] considering the Akaike information criteria (AIC) and the Bayesian information criterion (BIC) as the most reliable criteria. The AIC and BIC criteria were concordant in assigning the general time reversible (GTR + I + G) model to *cytb* as the best model of sequence evolution. BEAST v.1.8 [40] and MrBayes 3.2.6 [41] were used to generate a consensus tree based on coalescent Bayesian inference. BEAST was also used to obtain an estimate of the time to the most recent common ancestors (TMRCA). The choice of the right model could be challenging for such a data set; indeed the intraspecific framework could fall within a coalescent tree process which is more appropriate for a population-level analysis. However, the high levels of geographic structure and divergence between groups suggested that a Yule model would best fit the data. To infer the TMRCA, we assumed a relaxed molecular clock applying an uncorrelated lognormal distribution. Lacking a reliable calibration date on the root of the tree due to the absence of a fossil record, we applied a normal distribution to the mean-rate prior of the *cytb* mutation rate (μ = 0.0175). Since this substitution rate was the average of substitution rates found in *Podarcis* lizards for the gene *cytb*, the standard deviation of the normal prior distribution (SD = 0.0014) was set in order to encompass the minimum value of 0.0145 [42] and the maximum value of 0.02 [43] proposed for the genus *Podarcis*. We run three independent MCMCs of 100 million generation sampling every 10.000 steps and the program TRACER v.1.6 [44] was used to check for the convergence of the parameters. The Bayesian analysis performed using MrBayes was run using four heated, one million step chains with an initial burn-in of 100,000 steps. Both Bayesian analyses were performed using *Podarcis muralis* as outgroup (accession number HQ652936).

Statistical parsimony networks were constructed under 95% probability connection limits on each mtDNA clade identified by the phylogeny using TCS v.1.21 [45]. We used the same software to build additional further networks using the two nuDNA data sets.

Subsequently, we used a spatial principal component analysis (sPCA) as implemented in the R package ADEGENET v.1.2.8 [46] to explore the mtDNA spatial autocorrelation. The sPCA was applied to the whole data set and to each clade (see results for definition of the clades) to better highlight both the total and intraclade spatial distribution of the mtDNA. Unlike usual spatial analyses, eigenvalues of sPCA allow us to measure both the *cytb* genetic diversity (variance) and the spatial structure (spatial autocorrelation measured by Moran’s I). This decomposition can also be used to choose which principal component can be employed to test, based on Monte Carlo permutations (*N* = 9999), the presence of global and/or local structure [46]. All the 346 coordinates were jittered (randomly shifted) by a factor of 10 (about = 100 m) to avoid zero geographic distances between individuals from the same population. We used a Gabriel graph to generate a proximity connection network between populations. The sPCA variance was then plotted against the spatial autocorrelation (Moran’s I) to visualize the existence of global and local structure. Global scores can identify genetically distinguishable clusters, clines in allele frequencies and intermediate samples, while local scores can detect local differentiation between neighboring sites.

Finally, to explore the genetic structure of the nuclear markers, we used SPADS v.1.0 [47] to perform the analysis of molecular variance (AMOVA), testing the level of nuclear genetic diversity among groups, among populations and within populations grouped according to the mtDNA clades revealed by the phylogenetic analysis.

### Historical demography and spatial diffusion

Changes in population size over time were estimated by means of the multilocus Extended Bayesian Skyline Plot (EBSP) as implemented in BEAST v.1.8 [40]. The EBSP allows to combine information from different unlinked loci and thus to assess the uncertainty in the stochastic process (the coalescent), leading to improvement in the reliability of demographic inferences and a substantial reduction in estimating errors [48].

The EBSPs were performed maintaining the same prior settings used in the TMRCA analysis for the *cytb* (lognormal distribution; μ = 0.0175, SD = 0.0014), allowing the estimation of the nuclear genes.

The analysis was performed under a strict clock model, with unlinked substitution, clock and tree models for all the markers. We carried out three independent runs of 3 × 10^8^ generations sampled every 30^4^ intervals for each clade with a sample size >15, using either the multilocus (with or without the *β-fibint7*) or only the mtDNA data set. Since the multilocus analysis with the *β-fibint7* showed wider 95% highest posterior densities and large uninformative coalescent intervals due to the small sample size of this data set, we decided to eliminate this marker from the final runs. Conversely, the multilocus and mitochondrial EBSP yielded very similar results and we report only those from the multilocus analysis including the *mc1r*. The results were checked for diagnostic and parameters convergence using TRACER v.1.6 [44].

Finally, we used DnaSPv.5.1 to estimate Tajima’s *D* and Fu’s *Fs* statistics and to assess their significance through 10,000 coalescent simulations under the hypothesis of population equilibrium and selective neutrality.

To reconstruct the location of ancestral areas and spatial diffusion from these, we used a continuous Relaxed Random Walk model (RRW) in a Bayesian phylogeographic analysis (BPA) as implemented in BEAST v. 1.8 (Drummond et al. 2012). We performed separated analysis on each clade (with a sample size >15) using the mtDNA data set applying GMRF Bayesian skyride prior [49] and a Cauchy RRW model of diffusion across branches [50]. MCMC chains were run for 3 × 10^8^ generations sampling every 30^4^. Convergence of parameters for each run was assessed in TRACER v.1.6. The maximum clade credibility (MCC) was computed with TreeAnnotator v. 1.8. and was then used to visualize the spatial diffusion over time using the Time Slicer option in SPREAD v. 1.0.6 [51]. The 80% highest posterior densities (HPD) were estimated and plotted at three specific time intervals (2.3 Mya, 1.3 Mya and 0.1 Mya).

### Species distribution modelling

To better understand current and past habitat suitability of the species, we produced distribution models under different paleoclimatic conditions using species distribution models (SDM). The present-day occurrences of *P. siculus* were partially derived from the online database “Global Biodiversity Information System” (GBIF) and from the literature, but also from our georeferenced samples. The complete data set was trimmed to the spatial resolution of the environmental layers (2.5 arc min, about 4 km) to avoid duplications within the same cell.

To infer habitat suitability for the species, we evaluated the 19 climatic variables available from the Worldclim database [52]. The number of environmental layers was chosen on the basis of our knowledge of the ecology and habitat preferences of the species. Hence the following six variables were selected: mean diurnal range (BIO2), temperature seasonality (BIO4), minimum temperature of coldest month (BIO6), temperature annual range (BIO7), annual precipitation (BIO12) and precipitation seasonality (BIO15). Pearson correlation coefficients were then calculated to assess the level of autocorrelation among the selected variables. The level of autocorrelation was fairly low, with no pairwise comparison with *r* higher than 0.75.

The past distribution of the Italian wall lizard was generated using ensemble forecasting as implemented in the R package ‘biomod2’ [53]. We used four different algorithms which estimate species distributions using environmental predictors together with species occurrences: Maxent [54, 55], Gradient Boosting Machines (GBM) [56], Generalized Linear Model (GLM) [57] and General Additive Model (GAM) [58]. The four algorithms were used to predict the species occurrence for both the present-day and the last glacial maximum (LGM, ∼26–20 Kya). We randomly split the records into two subsets including 70% of records to calibrate the models and 30% for evaluation [53]. This procedure was repeated 10 times and for each replicate a random selection of 10,000 background points was performed including the entire extent of occurrence of *P. siculus*. The predictivity of each model was assessed with AUC and TSS criteria. We performed model averaging by weighting the individual model projections by their AUC or TSS scores, retaining only those models with AUC > 0.7 and TSS > 0.4 to avoid poorly calibrated models. Projection of the model to the LGM condition was obtained using both the Community Climate System Model (CCSM) and the Model for Interdisciplinary Research on Climate (MIROC) available in the Worldclim database.

## Results

### Phylogeographic structure and time of divergence

**Fig. 2:**
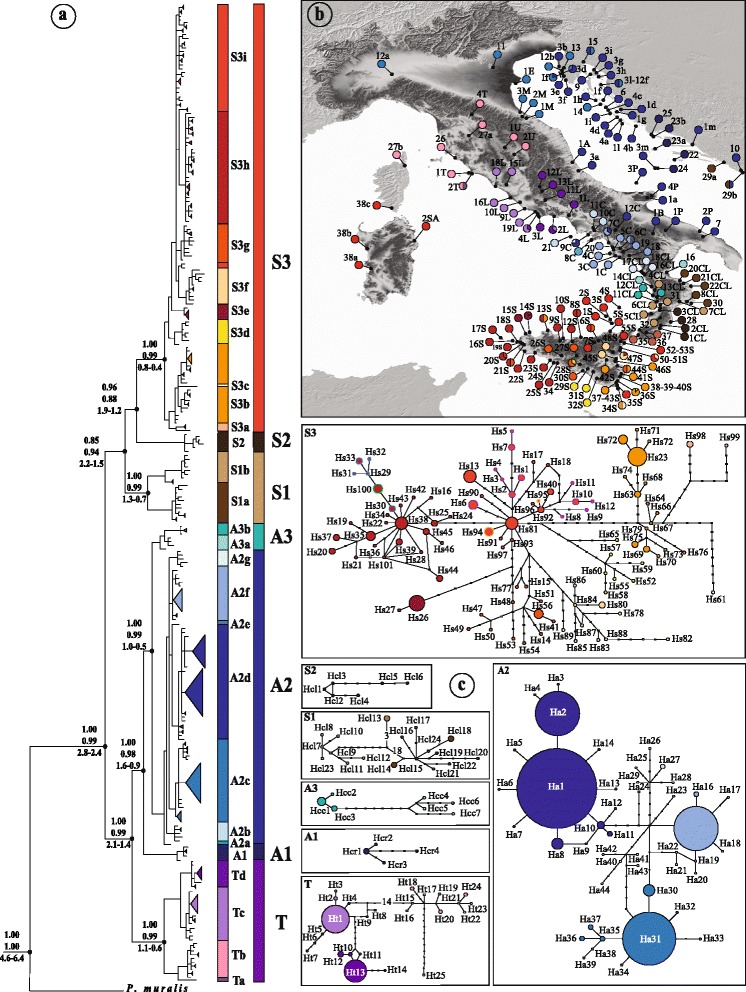
**a** Phylogenetic haplotype recostruction of the *cytb* data set obtained with MrBayes. At each node, MrBayes and BEAST posterior probability and TMRCA with 95% highest posterior density (HPD) intervals are respectively reported. The seven clade such as its nested haplogroups are depicted with different colors. **b** Geographic distribution of the mtDNA haplotypes in *Podarcis siculus*. Pie diagrams shows the haplotype frequencies at each sampled location and are colored according to the main haplogroups identified by the phylogeny. **c** Statistical parsimony networks of the seven mtDNA clades identified by the phylogeny. Each *circle* size is proportional to their frequencies and each *filled rectangles* representing one substitution. The different colors within each network depict the principal identified haplogroups. Circum-Sicilian and Corsica-Sardinia haplotypes are reported in *bold* with different colors (*violet*: Aeolian haplotypes; *blue*: Aegadian haplotypes, *green*: Sardinia and Corse haplotypes, *yellow*; Isola Bella haplotypes)

The final *cytb* alignment was 935 bp (*n* = 277) from our samples and 765 bp (*n* = 76) from Podnar et al. (2005). The coalescent Bayesian phylogenetic analysis, inferred using BEAST and MrBayes, supported the same topology with seven well-supported clades (see Fig. 2a for supporting values at each node): T, A1, A2, A3, S1, S2 and S3. We found high genetic divergence among clades, with an overall mean distance D_nei_ = 0.055, a maximum value between clade T and clade S1 (D_nei_ = 0.092) and a minimum value between clade A1 and A2 (D_nei_ = 0.028). These seven clades were also supported by sPCA, performed on the whole mtDNA (see below).

Estimates of the TMRCAshowed an early separation at 2.59 Mya (95% HPD: 2.78–2.39 Mya) into two main lineages, corresponding to the “Siculo-Calabrian” lineage and the “central-northern” lineage. Within the Siculo-Calabrian lineage we identified three clades (S1, S2 and S3). The emergence of S1 occurred at 1.9 Mya (95% HPD: 2.25–1.58 Mya) while S2 and S3 separated 1.54 Mya (95% HPD: 1.88–1.2 Mya). The central-northern lineage split approximately 1.79 Mya (95% HPD: 2.15–1.4 Mya) into two main groups that for simplicity we refer to as ‘Adriatic’and ‘Tyrrhenian’. The Adriatic group includes three clades with an early separation of the Croatian clade A1 at 1.22 Mya (95% HPD: 1.58 Mya–890 Kya) followed by a subsequent separation between clade A2 and the northern Calabrian clade A3 at 812 Kya (95% HPD: 1.03 Mya–552 Kya). See Fig. 2a for TMRCA at more internal nodes. We found a variable number of haplogroups within the seven clades. The haplogroups were defined by the high posterior probability support (0.97–0.99) in the phylogenetic analysis and by their clustering in the Parsimony network (Fig. 2). The Tyrrhenian clade T (*n* = 45, H =25) is mainly distributed across the northern-central Tyrrhenian coast and includes three principal haplogroups (Tb, Tc and Td) with a south–north pattern and a single diverging haplotype (Ht28). The TMRCA for this clade was estimated at 846 Kya (95% HPD: 1.14 Mya–573 Kya).

Clade A2 (*n* = 113, *H* = 44) is the only clade we found along the Adriatic coast (excluding clade A1 which is restricted to the Curzolan Islands, Croatia). It is characterized by five haplogroups and two unique diverging haplotypes. Two haplogroups and the two diverging haplotypes are restricted to Campania and Basilicata (A2a, A2b, A2e and A2f), one haplogroup is from northern Calabria (A2g) and the remaining two are widespread across the Adriatic coast of Italy and Croatia. The A2c haplogroup has the northernmost gravitation while the A2d haplogroup is widespread towards the Italian and Croatian Adriatic coasts. Both are characterized by a star-like shape and very low haplotype diversity.

Clade A3 (*n* = 10, *H* = 7) includes populations from northern coastal areas of Calabria (Catena Costiera). This clade is more related to the Adriatic clade A2, forming part of the central-northern lineage, than to the other clades found in southern Calabria.

Clades S1 (*n* = 22, *H* = 18) and S2 (*n* = 6, *H* = 6) represent the Calabrian “sensu stricto” lineages; in fact they are restricted to the Serre chain (and surrounding areas) and the Sila massif respectively. Clade S3 (*n* = 152, *H* = 101) groups haplotypes mainly belonging to Sicily. However, two southern Calabrian (Aspromonte massif) haplotypes (Hs98 and Hs99) also belong to this clade. They are separated by 10 and 13 substitutions from haplotypes found in the southernmost part of Sicily (Hyblean). Within Sicily, we identified 7 main haplogroups partially separated geographically (Fig. 2b-c) and a single divergent haplotype (Hs61) from Pachino. The oldest split within the clade was estimated at the Middle Pleistocene (95% HPD: 800 Kya–460 Kya). The southernmost region (Hyblean) is characterized by haplogroups S3b and S3c and the divergent haplotype from Pachino. Western Sicily mainly includes a single haplogroup (S3h) with the exception of two divergent haplotypes (Hs26 and Hs27) from the Zingaro Nature Reserve and Monte Cofano (haplogroup S3e). Central Sicily includes a principal haplogroup (S3g), while the north-eastern region includes haplogroup S3i which also comprises individuals from the Aeolian Islands and haplogroup S3f with circum-Etnean gravitation.

Among the circum-Sicilian archipelagos, the Aegadian Islands of Marettimo and Favignana (blue bold in Fig. 2c) appear to be monophyletic, while the Aeolian Islands (violet bold in Fig. 2c) are polyphyletic and thus appear to have experienced independent colonization events. However, we did not find any shared Aeolian and Sicilian haplotypes. It is noteworthy that individuals from southern Corsica and from central and north-western Sardinia (green bold in the Fig. 2c) fall into the sub-haplogroup present in the Aegadian Islands.

The sPCA, performed on the whole mtDNA, showed a stronger global than local structure (*P*_*global*_ < 0.001, *P*_*local*_ = 0.9). When only PC1 and PC2 were considered, the sPCA supported the separation of five clades (T, A, S1, S2 and S3), while PC3 better explained the variation within the Adriatic clades A1, A2 and A3. The sPCA performed within each clade indicated significant global rather than local structure in T, A2, A3, S1 and S3 (T: *P*_*global*_ = 0.001, *P*_*local*_ = 1; A2: *P*_*global*_ = 0.001, *P*_*local*_ = 1; A3: *P*_*global*_ = 0.014, *P*_*local*_ = 0.77; S3: *P*_*global*_ = 0.001, *P*_*local*_ = 1), while clade A1 did not show a significant value of either global or local structure (A1: *P*_*global*_ = 0.36, *P*_*local*_ = 0.61) and clade S2 showed higher local than global structure.

The final nuclear DNA alignments were726 bp (*n* = 182) for *β-fibint7*, and 600 bp (*n* = 524) for*mc1r*. No recombination events were found by the four-gamete and the phy test for both the nuclear genes.

**Fig. 3:**
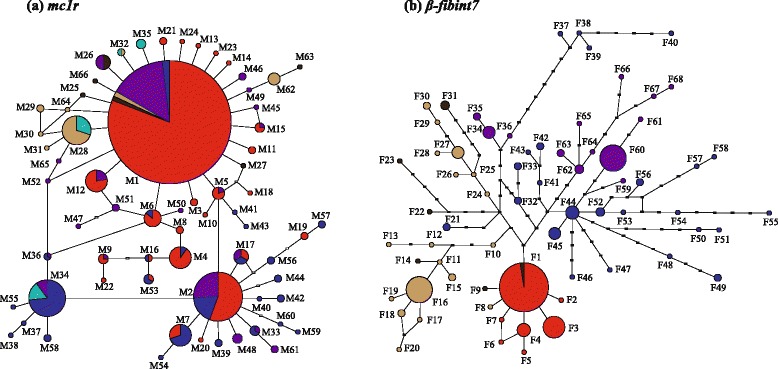
Statistical parsimony network connecting the 61 *mc1r* haplotypes (**a**) and the 68 *β-fibint7* haplotypes (**b**). Each haplotype is represented by *circles* with size proportional to their frequencies. The colors depicts the identified mitochondrial clades

We found 68 phased haplotypes for *β-fibint7* connected in a single network which showed a certain structure matching to the main mtDNA clades (Fig. 3). Estimates of genetic diversity showed high level of nucleotide (π = 0.009 ± 0.0005) and haplotype (*h* = 0.93 ± 0.01) diversity, remarked also by the hierarchical AMOVA which indicated that 40.3% (F_ct_ = 0.39) of variation was among groups (i.e., mtDNA clades), 46% (F_st_ = 0.45) was within populations and 13.7% (F_sc_ = 0.13) was among populations within groups. The geographic allele distribution of the main *β-fibint7* haplogroups is shown in Additional file 3: Figure S3.

The *mc1r* gene (consisting of 61 haplotypes) resulted less structured. The network connecting these haplotypes failed to identify clusters reflecting the lineages or the clades revealed by the mtDNA phylogenetic analysis (Fig. 3). Estimates of genetic diversity indicated lower values of nucleotide (π = 0.003 ± 0.0001) and haplotype (*h* = 0.69 ± 0.02) diversity compared to the *β-fibint7.* However, a certain level of geographic structure could be observed when the distribution of some haplotypes was taken into account (Additional file 4: Figure S4). We found the three most common haplotypes (M1, M2 and M34) present in 67% of the entire sample. They are connected to other low-frequency haplotypes in a star-like shape.

Haplotype M1 is widespread at high frequency in central Italy, Calabria and Sicily. M2 is found in all the studied area with the exception of Calabria. M34 is found in central-southern Italy and along the Adriatic coast, mirroring the distribution of the mtDNA ‘Adriatic’ clade. It is noteworthy that haplotype M1 was not found at all in this area. Finally, the analysis of molecular variance (AMOVA) carried out on *mc1r* using the grouping option revealed by the mtDNA clades indicated that 25.2% (F_ct_ = 0.87) of variation was among groups, 59% (F_st_ = 2.04) was within populations and 16% (F_sc_ = 0.55) was among populations within groups.

### Historical demography and spatial diffusion models

**Fig. 4:**
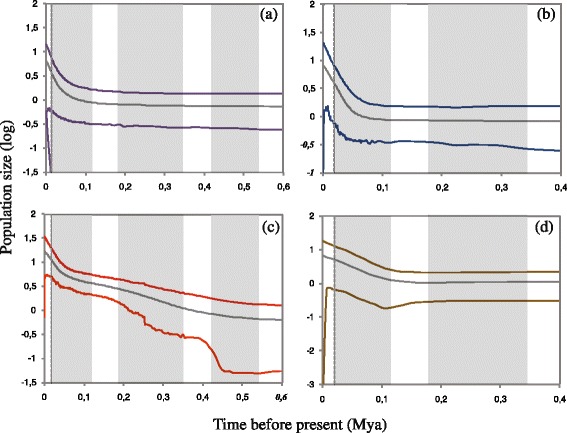
Extended Bayesian skyline plots inferred with BEAST, illustrating effective population sizes (Ne) through time. The *grey lines* represents the median population size, and the *colored lines* represent 95% higher posterior probability of (**a**) T clade, (**b**) A2 clade, (**c**) S3 clade and (**d**) S1 clade. The *grey rectangles* corresponds to the Würm, Riss and Mindel glacial periods while *dashed lines* indicate approximately the last glacial maximum

The historical demography was performed on clades T, A2, S1 and S3 which showed informative coalescent intervals in a time window of 0.6–0.4 Kya (Fig. 4). The results of the Tajima’s *D* and Fu’s *Fs* statistics are reported in Table 2.

**Table 2:** Mitochondrial (*cytb*) genetic diversity indices and neutrality tests for each clade

Clade	*n*	H	h ± SD	π ± SD	Tajima’s *D*	Fu’s *Fs*
T	45	25	0.94 ± 0.02	0.017 ± 0.001	0.27	−2.58
A1	5	4	1.00 ± 0.18	0.004 ± 0.001	\	\
A2	113	44	0.93 ± 0.01	0.0088 ± 0.0003	−1.45*	−20.34**
A3	10	7	0.96 ± 0.04	0.022 ± 0.003	0.13	0.136
S1	22	18	0.97 ± 0.02	0.023 ± 0.001	0.65	−2.36
S2	6	6	0.93 ± 0.12	0.0045 ± 0.001	\	\
S3	152	101	0.99 ± 0.002	0.0132 ± 0.0005	−1.76*	−84.9**

MtDNA sample size (*n*), number of haplotypes (H), haplotype diversity with standard deviation (h ± SD), nucleotide diversity with standard deviation (π ± SD), Tajima’s *D* and Fu’s *Fs* statistics for each identified clade

*P < 0.05 **P < 0.001

The EBSP of the Tyrrhenian clade T showed prolonged demographic stability and a gradual pre-LGM expansion starting about 80 Kya (Fig. 4a). The Adriatic clade A2 experienced a demographic expansion starting about 70 Kya (Fig. 4b). The EBSP of the Calabrian clade S1 showed prolonged stability followed by expansion occurred at the beginning of the last glacial phase (Fig. 4d). The Sicilian clade S3 experienced different demographic expansion events corresponding to the main glacial phases during the last 500 Kya (Fig. 4c).

**Fig. 5:**
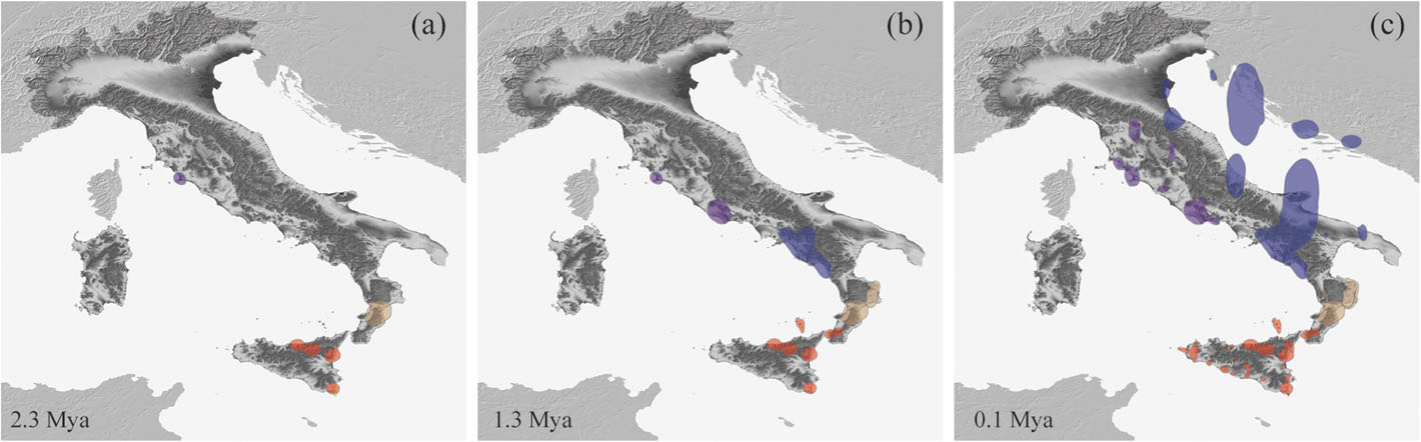
Estimates of spatial diffusion of *Podarcis siculus* using mtDNA data set at three time points during the Pleistocene (**a**) 2.3 Mya, (**b**) 1.3 Mya, (**c**) 0.1 Mya. The *polygons* are colored according to each clade and indicate the uncertainty (80% HPDs) surrounding geographic locations of internal nodes of the MCC tree

The Bayesian phylogeography using continuous RRW summarized in three time slices identified the presence of several ancestral areas with 80% HPDs at 2.3 Mya (Fig. 5). The Siculo-Calabrian lineage showed four ancestral areas in Sicily and one in Calabria while the central-northern lineage showed a single ancestral area located in the Tyrrhenian coast of Tuscany. At 1.3 Mya, the 80% HPDs identified the emergence of additional ancestral areas especially throughout the Tyrrhenian coast (which correspond to the location of the main haplogroups), from which population expanded up to achieve a maximal spatial diffusion at about 0.1 Mya.

### Species distribution modelling

**Fig. 6:**
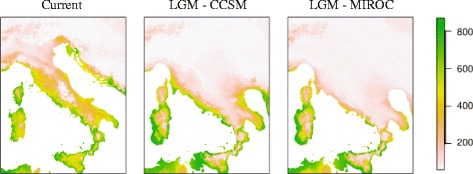
Species distribution model for *Podarcis siculus* indicating the present-day conditions and the last glacial maximum (LGM) based on the Model for Interdisciplinary Research on Climate (MIROC) and Community Climate System Model (CCSM)

Under current conditions (Fig. 6) the model predicts moderate and high suitability scores in coastal areas and in the southernmost regions but low habitat suitability in the whole Apennine mountain range and in northern regions. This fits very well with the known distribution of the species and its relative abundance.

Concerning the LGM prediction, high habitat suitability is maintained in coastal areas, especially throughout the Tyrrhenian coast and southern regions including a large area of high suitability in Sicily. Conversely, habitat suitability decreases in central and northern areas compared to the present-day distribution. The Adriatic Sea exhibited extensive land exposure due to the glacial marine regression. This area shows moderate to low habitat suitability along the coast and low habitat scores in internal areas.

## Discussion

The phylogeographic analysis of the Italian wall lizard confirmed previous studies showing a strong mitochondrial phylogeographic structure with multiple lineages across its range (Fig. 2) [30]. However, the extended sampling across the Italian Peninsula and Sicily allowed the identification of surprising geographically nested lineage diversity, with additional haplogroups not identified previously, reflecting a more complex genetic pattern. The number of mtDNA lineages and their high level of divergence are, to the best of our knowledge, among the highest ever observed in any vertebrate species from the area. A similar high number of mtDNA lineages can be observed in the congeneric *Podarcis muralis* [28]. However, in this species the genetic divergences among lineages is much lower than the one observed in *P. siculus*.

This very high genetic partioning allowed an uncommonly small geographic scale dissection of the effect of Pleistocene climatic oscillation across an entire Mediterranean Pleistocene refugial area. This interpretation reflects at least two aspects of genetic complexity associated with different time-scale processes. The first deep phylogeographic divergence corresponds to major geographic barriers which produced prolonged allopatric differentiation during the Early Pleistocene (2.6-0.7 Mya). A second minor phylogeographic discontinuity is observed within clades, mirroring independent local differentiation in response to climatic fluctuation during the Early-Middle Pleistocene transition. Finally, short-term demographic expansions corresponding to the last glacial phase were also observed.

### Long-term allopatric fragmentation and maintenance of the parapatry of clades

The structure of *P. siculus* mtDNA diversity into seven clades, with parapatric distribution, is strongly supported by both the phylogenetic analysis and sPCA, indicating significant global structure partioned into genetically distinguishable clusters.

Although the intrinsic inaccuracy of the molecular clock has been repeatedly reported ([59–62] and references therein), suggesting caution in the use of genetic data to infer dates of historical events, our approximate time window of long-term allopatric fragmentation appears to agree well with the major geomorphological events which characterized the main tectonic arrangements and eustatic events of the Italian Peninsula. The most ancient separation during the Pliocene-Pleistocene transition (approximately 2.78–2.39 Mya) matches the split of the southernmost Siculo-Calabrian lineage from the central-northern lineage. This result based on mtDNA is in accordance with an allozyme analysis at twenty loci [63]. The location of this genetic discontinuity reflects the specific position of the Crati-Sibari plain in northern Calabria. Indeed, this area acted as a dispersion barrier during the repeated Plio-Pleistocene marine transgressions separating the Italian Peninsula from the Calabrian and Sicilian paleo-archipelagos [64, 65]. This main vicariance event has also been observed in other detailed works on different vertebrate taxa, confirming this region as one of the most important in driving long-term allopatric differentiation in southern Italy [25, 66–69].

The more recent split of the three clades within the Siculo-Calabrian lineage, dated approximately 1.54–1.9 Mya, probably reflects the Early Pleistocene fragmentation of the Calabrian arc into paleo-islands corresponding to the Serre and Sila massifs and Aspromonte. In fact, clade S1 is found in the Sila massif and surrounding areas, while S2 is mainly distributed across the Serre region. It is interesting to note that an overlapping phylogeographic structure has been reported in the region for the Italian newt, *Lissotriton italicus* and the Roman mole, *Talpa romana* [25, 69].

The presence of an ancient S3 clade mostly distributed throughout Sicily is not straightforward, having few paralellisms in the literature [70–72]. This continental island experienced recurrent connection with the mainland during the glacial phases. It should be pointed out that the S3 clade and some sicilian *β-fibint7* haplotypes were also found in the southernmost part of Calabria. According to the BPA this occurrence suggested an ancient backward recolonization event probably due to land bridge connections during the Gunz-Mindel transition. The more ancient Middle Pleistocene split within Sicilian haplogroups agrees geographically and temporally with the presence of the Hyblean and Peloritani land masses, which were separated by the Gela-Catania channel until the more recent emergence of the Etna Volcano [73, 74]. This dichotomy is also evident in the position of the ancestral areas in Sicily depicted by the BPA (Fig. 5a).

Deep vicariance events during the Early-Middle Pleistocene (2.25–1.58 Mya) were also observed between the T and A2 clades within the central-northern lineage. The geographic boundary observed between the Tyrrenian clade T and the Adriatic clade A2, whose split is dated at 1.9 Mya (Early Pleistocene), has been reported in various taxa [20, 28, 75]. The genetic discontinuites seem to be located in different geographic positions. The south–north boundary corresponds to a well-known deep phylogeographic discontinuity matching the Volturno plain. This area was repeatedly flooded by Middle Pleistocene marine transgressions, thus constituting an effective geographic barrier between previously separated populations [76, 77]. Conversely, the west–east boundary seems to be the outcome of a recent recolonization route of the A2d haplogroup from the southernmost regions (see below), confirming the role of the Apennine range as a longitudinal diffusion barrier.

While the *β-fibint7* showed evidence of genetic discontinuity according to the mitochondrial signal, the *mc1r* resulted in a lower phylogeographic structure. The occurrence of incomplete lineage sorting to explain mito-nuclear discordance has been describer for an increasing number of case studies [78–80]. Thus, the failure of nuclear genome to diverge as a consequence of its slower diverging rate and higher population size appear the most plausible interpretation to explain such pattern rather than sex-biased gene flow [81–83]. Moreover, the higher number of retained polymorphisms in the *mc1r* marker is also consistent with the more relaxed rate of lineage sorting for coding genes which therefore could be under selection [84–86]. However, the two nuclear genes also showed a certain degree of consistency regarding the locations of the principal genetic discontinuities (Additional file 3: Figure S3 and Additional file 4: Figure S4). This is remarked by the high percentage, for both genes, of the among groups variation (40.3% for *β-fibint7* and 25.2% for *mc1r*) revealed by the AMOVA, hierarchically defined by the mtDNA clades.

One of the main questions emerging from this study is how such close parapatric structure between mtDNA clades, as seen in Fig. 2b, could be maintained. This pattern is also supported by the complete absence (out 59 localities with 2 < N < 10) of the co-occurrence of mtDNA clades in the same locality, even though the outcome of repeated cycles of allopatric fragmentation and local expansion would give rise to a certain admixture of genetic lineages. This phenomenon is only now becoming better known [87, 88] and the main explanations include “density blocking”, local adaptation (such as adaptation of the mtDNA genome) and competitive exclusions. In this case-study, we cannot exclude a certain level of hybrid unfitness [87]. In fact, the level of genetic divergence at *cytb* is only slightly lower than that commonly found between sister species in *Podarcis* [42, 89, 90].

Further detailed studies are required in the contact areas of the mtDNA clades, including estimates of gene flow using more variable markers (i.e. microsatellites) and detailed analyses of the sexual selection and other eco-ethological characteristics of these differentiated interacting populations [91, 92].

### Dissecting the evolutionary history and demography of the clades

The evolutionary history of the clades appears to have been much more influenced by a series of historical fragmentations due to severe cycles of glacial/interglacial periods during the Early-Middle Pleistocene than by recent demographic responses to the LGM. However, we also found independent responses of each clade to such long-term and short-term processes.

The main minor genetic discontinuities within clades are traceable to a time window corresponding to the Early-Middle Pleistocene transition (1.3–0.7 Mya) [93]. The latter subepoch transition, also known as the ‘Mid-Pleistocene Revolution’ [94], is associated with a series of complex climatic changes leading to a more severe cold stage [95]. The main outcome of the increasing amplitude in climatic fluctuations is the more drastic responses of temperate species in retreating into suboptimal refugial areas. This phenomenon is well exemplified by clades S2 and T. The separation of the two main haplogroups in clade S2 reflects two possible refugial areas corresponding to the northern and southern areas surrounding the Sila massif, which finds some parallelisms in the literature [25, 69]. The BPA identified two putative refugial areas, well apart along the Tyrrhenian coast, for the haplogroups within the clade T. It is noteworthy that the current distribution of the two main haplogroups (Tc and Td) across this central peninsular region appears very similar to that observed in the southern smooth newt *Lissotriton vulgaris meridionalis* [29].

A different scenario emerges from examination of the genetic pattern in Sicily (clade S3). Since there are no substantial geographic barriers across the entire island, the strong phylogeographic structure, with seven haplogroups, is quite surprising and provides the highest resolution to date for the structure of Sicilian phylogeographic diversity [72]. At least two considerations deserve attention regarding this insular context. First, the temporal incongruence of the intra-island diversification (late Middle Pleistocene) with the Early-Middle Pleistocene transition could be the outcome of attenuated or shifted effects of the Pleistocene oscillation throughout this southernmost Mediterranean island. In any case, the perception that Sicily has experienced reduced climate-driven environmental changes is also evident by comparing current and past SDM. Second, the low variation of the nuclear markers suggests that these haplogroups would not have had completely independent evolutionary histories, maintaining a long-term mutation drift equilibrium. This is also confirmed by the SDM indicating different stable connected areas that may have guaranteed this gene exchange and equilibrium.

The cradle of clade A2 appears to be in the southern part of its distribution, as indicated by the BPA, where five of the seven haplogroups are located. Within this clade we found a combination of ancient isolation and more recent demographic dynamics. The main genetic discontinuities are restricted to southern-central Italy and may reflect Middle Pleistocene fragmentation due to the complex topography of this area. However, haplogroups A2d and A2c show a north-eastern gravitation, being the only haplogroups found on the Adriatic coast and in the Po Valley. The demographic inference for this clade, supported by the BPA, highlights an overall population expansion during the last glacial phase (about 70 Kya) from localities settled in the southern Tyrrhenian coast. Thus the presence of this clade throughout the Croatian coast is not very surprising considering the extended Adriatic land exposure during this period [96]. A similar glacial Adriatic expansion has also been proposed for *Hyla intermedia* [66]. Furthermore, such glacial colonization would have taken place from just a few pioneers, as suggested by the very low genetic diversity encountered and the overall low LGM habitat suitability predicted for this area.

Finally, while a vast body of literature clearly demonstrates that many terrestrial species underwent large and sudden latitudinal shifts following the end of the last glacial maximum (LGM) approximately 20 Kya ([1, 3, 97] and refs therein), we found a different scenario consistent with glacial expansions or regional persistence during the LGM. The hypothesis of glacial expansion and local persistence, reported in a growing number of Palearctic and Nearctic species [28, 98, 99], was criticized for the possible inaccuracy of molecular clock rates [100, 101]. However, our claim is supported not only by the estimated time of divergence but also by the evidence of large amount of new environment with high suitability, predicted by SDM, which appear during LGM in different southern Italian regions. This is particularly evident for the Sicilian clade S3. Indeed our demographic scenario indicates two expansions corresponding to the main glacial phases at 400 Kya and 100 Kya. As a matter of fact, the SDM prediction at LGM shows that Sicily is the area where the effect of the increase of high suitability areas and lowland exposure is most prominent for the entire species range. Although the glacial expansions in T and S2 were of smaller strength, these areas were also influenced by the increase of the coastal plain during the last glacial phase.

## Conclusions

### The exception became the rule?

The main outcomes of this study show that the classic EC model Pleistocene biogeography may be a far too simplistic interpretation of the evolutionary history of *P. siculus*. The high number of deeply divergent clades matches a ‘refugia within refugia’ scenario [1] rather than a single panmictic refugium for the Italian Peninsula. However, despite the greater number of clades located in southern regions as expected from the “southern richness” scenario [1, 2, 102], our results showed a genetic structure conforming more to a pattern that here can be called “western richness and eastern purity”. This scheme also appears to be well supported by the current and past SDM predictions and location of ancestral areas indicated by BPA. In fact, the Tyrrhenian coastline appears to have maintained more stable climatic conditions than the Adriatic one. Hence, the southernmost and Tyrrhenian regions represent hot pockets in which populations may have preserved their deep genetic integrity by persisting in scattered refugial areas during strong Pleistocene cooling periods, from which they later could have expanded towards new areas. We did not find evidence of expansion subsequent to the LGM in any of the analyzed clades. Instead we found genetic signatures of demographic expansion preceding the LGM or long periods of stability. This complex assembly of genetic signatures appears to be distant from the expectation of the EC models and post-LGM recolonizations, becoming one of an increasing number of cases in which these assumptions are not met.

The historical demography coupled with the evolutionary history reconstruction indicated that long-term fragmentation and/or pre-LGM events such as glacial persistence were more important in shaping the genetic architecture in this temperate species. Such historical events may have influenced populations to different extents, indicating the importance of local topographic impacts on Quaternary climatic oscillations in inducing population-specific responses to the ever changing environmental variability.

## Additional files

Additional file 1: Table S1. Sample size of each locality (with relative locality codes). MtDNA and nuDNA haplotypes are also reported for each sampling location. (DOCX 54 kb)
Additional file 2: Table S2. Voucher with relative accesison numbers and haplotypes. (DOCX 58 kb)
Additional file 3: Figure S3. Geographic allele distribution of *β-fibint7* with relative frequencies indicated by pie diagrams at each sampled location. (JPG 4081 kb)
Additional file 4: Figure S4. Geographic allele distribution of *mc1r* with relative frequencies indicated by pie diagrams at each sampled location. (JPG 4161 kb)

## Abbreviations

AIC: Akaike information criterion
AUC: Area under the curve
BIC: Bayesian information criterion
BPA: Bayesian phylogeographic analysis
CCSM: Community climate system model
EBSP: Extended Bayesian Skyline Plot
EC: Expansion-contraction
GAM: General additive model
GBIF: Global biodiversity information system
GLM: Generalized linear model
HPD: Highest posterior distribution
IUPAC: International union of pure and applied chemistry
Kya: Thousand (10^3^) years ago
LGM: Last glacial maximum
MCC: Maximum clade credibility
MCMC: Markov chain Monte Carlo
MIROC: Model for interdisciplinary research on climate
mtDNA: Mitochondrial DNA
Mya: Million (10^6^) years ago
nuDNA: Nuclear DNA
PCA: Principal component analysis
RRW: Relaxed random walk
SDM: Species distribution modelling
TMRCA: Time of the most recent common ancestor
TSS: True skill statistic
