# Supplementary material for: A combination of long term fragmentation and glacial persistence drove the evolutionary history of the Italian wall lizard Podarcis siculus
Source: BMC Evol Biol. 2017 Jan 5;17:6. doi: 10.1186/s12862-016-0847-1 (PMC5216540; doi:10.1186/s12862-016-0847-1)
Supplement: Additional file 1: Table S1. — Sample size of each locality (with relative locality codes). MtDNA and nuDNA haplotypes are also reported for each sampling location. (DOCX 54 kb) [file 12862_2016_847_MOESM1_ESM.docx]

| Locality code | Locality | Lat | Long |  | mtDNA *cytb* | |  | nuDNA *mc1r* | | | nuDNA *β-fibint7* | |
| --- | --- | --- | --- | --- | --- | --- | --- | --- | --- | --- | --- | --- |
|  |  |  |  | n | Clade | Haplotype(s) | | | n | Haplotype(s) | n | Haplotype(s) |
| 1aS | Vulcanello | 38,421 | 14,959 | 2 | S3 | Hs1(1), Hs2(1) | | | 8 | M1(6), M2(2) | \ | \ |
| 1bS | Vulcano | 38,403 | 14,950 | 4 | S3 | Hs1(1), Hs2(1), Hs3(1), Hs4(1) | | | 10 | M1(2), M2(4), M3(1), M4(2), M5(1) | \ | \ |
| 2aS | Salina Malfa | 38,580 | 14,833 | 3 | S3 | Hs5(1), Hs6(1), Hs7(1) | | | 6 | M1(2), M2(1), M4(1), M6(1), M7(1) | \ | \ |
| 2bS | Salina Pollara | 38,578 | 14,806 | 1 | S3 | Hs68(1) | | | 2 | M1(2) | \ | \ |
| 3S | Lipari | 38,454 | 14,955 | 2 | S3 | Hs6(1), Hs9(1) | | | 4 | M1(4) | \ | \ |
| 4S | Stromboli | 38,808 | 15,229 | 6 | S3 | Hs6(1), Hs7(1), Hs10(2), Hs11(1), Hs12(1) | | | 10 | M1(5), M2(2), M4(1), M8(1), M9(1) | \ | \ |
| 5S | Capo D’orlando | 38,152 | 14,760 | 1 | S3 | Hs13(1) | | | 2 | M2(1),M10(1) | \ | \ |
| 6S | Nebrodi | 37,874 | 14,626 | 4 | S3 | Hs13(2), Hs14(1), Hs15(1) | | | 4 | M1(2), M2(1), M5(1) | 4 | F1 (3), F2(1) |
| 7S | Capizzi | 37,809 | 14,475 | 1 | S3 | Hs13(1) | | | 2 | M1(2) | \ | \ |
| 8S | Pollina | 38,011 | 14,144 | 4 | S3 | Hs16(1), Hs17(1), Hs18(1), Hs19(1) | | | 8 | M1(7), M4(1) | \ | \ |
| 9S | Roccapalumba | 37,825 | 13,637 | 2 | S3 | Hs20(2) | | | 4 | M1(1), M2(1), M11(2) | \ | \ |
| 10S | Termini Imerese | 37,965 | 13,734 | 1 | S3 | Hs21(1) | | | 2 | M1(1), M4(1) | \ | \ |
| 11S | Cerda | 37,927 | 13,792 | \ | S3 | \ | | | 2 | M1(1), M2(1) | \ | \ |
| 12S | Caltavuturo | 37,831 | 13,890 | 1 | S3 | Hs22(1) | | | 2 | M1(2) | \ | \ |
| 13S | Sferrocavallo | 38,210 | 13,288 | 3 | S3 | Hs23(1), Hs24(1), Hs25(1) | | | 4 | M1(4) | \ | \ |
| 14S | Zingaro | 38,094 | 12,798 | 6 | S3 | Hs26(4), Hs27(1), Hs28(1) | | | 14 | M1(11), M2(1), M12(2) | 8 | F1(8) |
| 15S | Monte Cofano | 38,105 | 12,677 | 1 | S3 | Hs26(1) | | | 2 | M1(1)-M13(1) | \ | \ |
| 16S | Favignana | 37,915 | 12,320 | 5 | S3 | Hs29(1), Hs30(2), Hs31(1), Hs32(1) | | | 12 | M1(11), M2(1) | 4 | F1(1), F4(3) |
| 17S | Marettimo | 37,970 | 12,071 | 2 | S3 | Hs33(2) | | | 4 | M1(4) | 4 | F1(4) |
| 18S | Stagnone | 37,866 | 12,486 | 4 | S3 | Hs34(1), Hs35(2), Hs36(1) | | | 8 | M1(8) | \ | \ |
| 19S | Rilievo Fiume | 37,884 | 12,528 | 5 | S3 | Hs35(1), Hs37(2), Hs38(2) | | | 10 | M1(10) | 2 | F3(2) |
| 20S | Borgo Fazio | 37,854 | 12,714 | 3 | S3 | H35(1), Hs39(1), Hs40(1) | | | 6 | M1(6) | \ | \ |
| 21S | Fiume Balata | 37,872 | 12,604 | 2 | S3 | Hs39(1), Hs41(1) | | | 6 | M1(5), M14(1) | \ | \ |
| 22S | Mazara | 37,707 | 12,652 | 2 | S3 | Hs38(1), Hs42(1) | | | 2 | M1(4) | 2 | F1(2) |
| 23S | Menfi | 37,586 | 12,997 | 1 | S3 | Hs43(1) | | | 2 | M1(2) | 2 | F1(1), F4(1) |
| 24S | Eraclea Minoa | 37,384 | 13,306 | 1 | S3 | Hs38(1) | | | 2 | M1(1), M15(1) | 2 | F1(2) |
| 25S | Siculiana | 37,341 | 13,391 | 4 | S3 | Hs44(1), Hs45(2), Hs46(1) | | | 6 | M1(6) | \ | \ |
| 26S | Milena | 37,482 | 13,710 | 4 | S3 | Hs47(1), Hs48(1), Hs49(1), Hs50(1) | | | 8 | M1(6), M7(1), M16(1) | \ | \ |
| 27S | Caltanissetta | 37,435 | 14,100 | 1 | S3 | Hs51(1) | | | 2 | M1(2) | \ | \ |
| 28S | Mazzarino | 37,311 | 14,190 | 3 | S3 | Hs52(1), Hs53(1), Hs54(1) | | | 6 | M1(4), M7(1), M15(1) | \ | \ |
| 29S | L. Disueri | 37,435 | 14,100 | 1 | S3 | Hs55(1) | | | 2 | M1(2) | \ | \ |
| 30S | Manfria | 37,105 | 14,157 | 3 | S3 | Hs56(3) | | | 2 | M1(2) | \ | \ |
| 31S | Sughereta | 37,071 | 14,439 | 3 | S3 | Hs57(1), Hs58(1), Hs59(1) | | | 12 | M1(8), M2(1), M4(1), M9(1)-M17(1) | \ | \ |
| 32S | Vittoria | 36,968 | 14,550 | 1 | S3 | Hs60(1) | | | 2 | M1(1), M2(1) | \ | \ |
| 33S | Ispica | 36,793 | 14,881 | \ | S3 | \ | | | 4 | M4(1), M18(1), M19(2) | \ | \ |
| 34S | Pachino | 36,731 | 15,007 | 2 | S3 | Hs23(1), Hs61(1) | | | 4 | M1(4) | \ | \ |
| 35S | Porto Palo | 36,716 | 15,121 | 1 | S3 | Hs40(1) | | | 2 | M1(2) | 2 | F1(2) |
| 36S | Vendicari | 36,803 | 15,096 | 10 | S3 | Hs23(2), Hs62(1), Hs63(2), Hs64(1), Hs65(1), Hs66(1), Hs67(1), Hs68(1) | | | 18 | M1(9), M4(1), M7(1), M9(1), M12(3), M17(1), M20(1), M21(1) | 10 | F1(9), F7(1) |
| 37S | Noto Lido | 36,847 | 15,110 | 3 | S3 | Hs69(2), Hs70(1) | | | 6 | M1(3), M2(2), M6(1) | \ | \ |
| 38S | Cavagrande | 36,985 | 15,030 | 1 | S3 | Hs71(1) | | | 2 | M1(1), M2(1) | \ | \ |
| 39S | Cassibile | 36,982 | 15,165 | 2 | S3 | Hs72(2) | | | 4 | M1(3), M12(1) | 2 | F4(1), F6(1) |
| 40S | Canicattini | 37,064 | 15,122 | 3 | S3 | Hs23(2), Hs72(1) | | | 6 | M1(4), M5(1), M6(1) | \ | \ |
| 41S | Florida | 37,117 | 15,141 | 1 | S3 | Hs73(1) | | | 2 | M1(2) | \ | \ |
| 42S | Saline di Priolo | 37,153 | 15,180 | 2 | S3 | Hs74(1), Hs75(1) | | | 4 | M1(4) | 4 | F1(3), F5(1) |
| 43S | Sorciano | 37,160 | 15,159 | 1 | S3 | Hs75(1) | | | 2 | M1(2) | \ | \ |
| 44S | Lentino | 37,334 | 14,999 | 2 | S3 | Hs76(1), Hs77(1) | | | 4 | M1(3)-M12(1) | 2 | F3(2) |
| 45S | Foce Simeto | 37,378 | 15,035 | 1 | S3 | Hs78(1) | | | 2 | M1(2) | \ | \ |
| 46S | Corridore del pero | 37,321 | 15,045 | 1 | S3 | Hs79(1) | | | 2 | M1(2) | \ | \ |
| 47S | Acireale | 37,598 | 15,172 | 5 | S3 | Hs80(2), Hs81(1), Hs82(1), Hs83(1) | | | 10 | M1(8), M2(1), M6(1) | \ | \ |
| 48S | Trecastagne | 37,621 | 15,091 | 3 | S3 | Hs84(1), Hs85(1), Hs86(1) | | | 6 | M1(4), M6(1), M22(1) | \ | \ |
| 49S | Pedara | 37,617 | 15,048 | 1 | S3 | Hs87(1) | | | \ | \ | \ | \ |
| 50S | Scura | 37,664 | 15,155 | 1 | S3 | Hs88(1) | | | 2 | M1(1), M2(1) | \ | \ |
| 51S | Pozzillo | 37,672 | 15,193 | 5 | S3 | Hs81(2), Hs89(1), Hs90(1), Hs91(1) | | | 12 | M1(7), M2(3), M6(1), M23(1) | \ | \ |
| 52S | Isola bella (spiag.) | 37,851 | 15,298 | 2 | S3 | Hs92(1), Hs93(1) | | | 6 | M1(4), M6(1), M24(1) | 2 | F1(1), F3(1) |
| 53S | Isola bella | 37,850 | 15,300 | 4 | S3 | Hs94(3), Hs95(1) | | | 8 | M1(6), M2(1), M15(1) | 8 | F1(4), F3(4) |
| 54S | Gaggi | 37,861 | 15,210 | 1 | S3 | Hs92(1) | | | 2 | M1(1)-M2(1) | \ | \ |
| 55S | Gole Alcantara | 37,872 | 15,166 | 3 | S3 | Hs81(1), Hs96(1), Hs97(1) | | | 8 | M1(8) | 4 | F1(2), F3(1), F4(1) |
| 1CL | Gerace | 38,276 | 16,220 | 2 | S2 | Hcl1(1), Hcl5(1) | | | 4 | M1(1), M25(1), M26(2) | 4 | F8(1), F22(1), F22(1), F31(1) |
| 2CL | Mammola | 38,364 | 16,243 | 2 | S2 | Hcl2(1), Hcl3(1) | | | 4 | M1(2), M26(1), M27(1) | 4 | F1(1),F14(1), F31(2) |
| 3CL | Maida | 38,858 | 16,386 | 1 | S2 | Hcl6(1) | | | \ | \ |  |  |
| 4CL | Rende | 39,331 | 16,185 | 2 | S2 | Hcl8(1), Hcl10(1) | | | 4 | M28(2), M29(1), M30(1) | 4 | F16(1), F24(1), F30(2) |
| 5CL | Mileto | 38,608 | 16,061 | 2 | S2 | Hcl13(2) | | | 4 | M1(3)-M31(1) | 4 | F16(1), F17(1), F20(1), F29(1) |
| 6CL | Vena di Maida | 38,859 | 16,369 | 1 | S2 | Hcl7(1) | | | 2 | M1(1)-M28(1) | 2 | F9(1), F27(1) |
| 7CL | Simeri-Crichi foce | 38,849 | 16,678 | 3 | S2 | Hcl9(1), Hcl11(1), Hcl12(1) | | | 4 | M28(3), M29(1) | 6 | F15(1), F16(1), F18(1), F26(1), F27(1), F28(1) |
| 8CL | Steccato di Cutro | 38,938 | 16,910 | 2 | S2 | Hcl19(1), Hcl20(1) | | | 2 | M28(1), M32(1) | 4 | F16(4) |
| 11CL | Falerna | 39,000 | 16,178 | 1 | A3 | Hcc3(1) | | | \ | \ | \ | \ |
| 12CL | Altilia (su.A3 per) | 39,130 | 16,256 | 3 | A3 | Hcc1(3) | | | 6 | M1(2), M32(1), M35(2) | 6 | F10(1),F12(1),F13(1),F15(1),F25(1) F52(1) |
| 13CL | Belmonte | 39,161 | 16,083 | 2 | A3 | Hcc2(1), Hcc3(1) | | | 2 | M28(2) | \ | \ |
| 14CL | Fiumefreddo | 39,232 | 16,075 | 3 | A3 | Hcc4(1), Hcc5(1), Hcc6(1) | | | 6 | M1(1), M28(2)-M35(1) | 6 | F16(2), F27(4), |
| 16CL | Cirella | 39,699 | 15,802 | 1 | A2 | Ha40(1) | | | 6 | M34(2), M36(2), M37(1), M38(1) | \ | \ |
| 17CL | Scalea | 39,820 | 15,793 | 1 | A2 | Ha44(1) | | | 2 | M2(1),M34(1) | \ | \ |
| 18CL | Praia a Mare | 39,889 | 15,786 | 2 | A2 | Ha43(1), Ha42(1) | | | 4 | M33(2), M34(2) | \ | \ |
| 20CL | Rossano | 39,578 | 16,635 | 3 | A2 | Hcl14(2), Hcl15(1) | | | 10 | M1(3), M6(1), M29(2), M62(3), M65(1) | 2 | F11(1), F19(1) |
| 21CL | Marina di Mandatorriccio | 39,537 | 16,866 | 1 | A2 | Hcl21(1) | | | 4 | M1(1), M21(1), M62(1), M64(1) | \ | \ |
| 22CL | Cirò marina | 39,361 | 17,126 | 3 | A2 | Hcl16(1), Hcl17(1), Hcl22(1) | | | 8 | M1(2), M28(2), M29(1), M62(1), M63(1), M66(1) | 2 | F16(3), F18(1) |
| 1P | Gravina | 40,796 | 16,423 | 1 | A2 | Ha9(1) | | | \ | \ | 2 | F21(2) |
| 2P | Rosa Marina | 40,793 | 17,556 | 1 | A2 | Ha6(1) | | | 2 | M2(2) | \ | \ |
| 3P | S. Domino | 42,117 | 15,494 | 1 | A2 | Ha12(1) | | | 2 | M2(1), M39(1) | 2 | F42(2) |
| 4P | Foresta Umbra | 41,823 | 15,954 | 6 | A2 | Ha1(5), Ha2(1) | | | 8 | M2(2), M4(1), M40(1), M41(1) M43(1), M44(2) | 6 | F41(1), F43(1), F46(1), F47(1), F50(1), F51(1) |
| 1C | Rovine di Velia | 40,162 | 15,164 | 5 | A2 | Ha15(4), Ha17(1) | | | 8 | M34(8) | 6 | F52(2), F56(2), F57(1), F58(1) |
| 3C | Pioppi | 40,234 | 14,945 | 1 | A2 | Ha22(1) | | | 2 | M34(1), M55(1) | \ | \ |
| 4C | Punta Licosa | 40,234 | 14,945 | 4 | A2 | Ha15(1), Ha19(1), Ha20(1), Ha21(1) | | | 8 | M1(1), M34(3), M42(1), M56(2), M57(1) | 4 | F44(2), F45(2) |
| 5C | Capaccio | 40,417 | 15,095 | 2 | A2 | Ha15(2) | | | 4 | M2(1), M34(1), M57(1), M58(1) | \ | \ |
| 6C | Roscigno | 40,414 | 15,335 | 2 | A2 | Ha15(1), Ha24(1) | | | 4 | M2(1), M34(2), M58(1) | 2 | F44(1), F45(1) |
| 7C | R.N. Sale Tanagro | 40,520 | 14,924 | 4 | A2 | Ha15(1), Ha16(2), Ha5(1) | | | \ | \ | 4 | F39(1), F45(3) |
| 8C | Amalfi | 40,636 | 14,682 | 1 | A2 | Ha23(1) | | | \ | \ | \ | \ |
| 9C | Ercolano | 40,799 | 14,352 | 4 | A2 | Ha10(3), Ha29(1) | | | \ | \ | 2 | F37(1), F48(1) |
| 10C | Palma Campania | 40,868 | 14,562 | 3 | A2 | Ha27(2), Ha28(1) | | | 6 | M17(1), M34(5) | 4 | F38(1), F40(1), F49(2) |
| 11C | Liveri | 40,901 | 14,582 | 1 | A2 | Ha26(1) | | | 2 | M34(2) | \ | \ |
| 12C | Conza | 40,841 | 15,291 | 2 | A2 | Ha11(2) | | | 4 | M2(1), M6(1), M39(1), M59(1) | 4 | F44(1), F53(1), F54(1), F55(1), |
| 1B | Melfi | 40,983 | 15,648 | 1 | A2 | Ha11(1) | | | 2 | M2(1), M5(1) | \ | \ |
| 1L | Suio Terme | 41,296 | 13,868 | 1 | T | Ht10(1) | | | 2 | M1(1), M34(1) |  |  |
| 2L | Sperlonga | 41,261 | 13,448 | 3 | T | Ht1(1), Ht10(1), Ht13(1) | | | 6 | M1(1), M2(1), M21(1), M34(1), M45(1), M46(1) | \ | \ |
| 3L | Circeo foresta | 41,355 | 13,058 | 1 | T | Ht12(1) | | | 2 | M1(2) | \ | \ |
| 4L | Lago dei Monaci | 41,405 | 12,876 | 6 | T | Ht1(3), Ht4(1), Ht12(2) | | | 10 | M1(7), M26(2), M34(1) | 4 | F60(2), F64(1), F66(1) |
| 9L | Foglino | 41,473 | 12,716 | 6 | T | Ht1(2), Ht3(1), Ht5(1), Ht8(1), Ht9(1) | | | 14 | M1(9), M47(1), M48(3), M49(1) | 8 | F34(1), F59(1), F60(2), F62(2), F67(1), F68(1) |
| 10L | Castelporziano | 41,706 | 12,419 | 2 | T | Ht2(2) | | | 4 | M12(1), M15(1), M50(1), M51(1) | 4 | F34(2), F60(2) |
| 11L | Anagni | 41,738 | 13,161 | 2 | T | Ht13(2) | | | 4 | M1(3)-M2(1) | \ | \ |
| 12L | Bellegra | 41,878 | 13,030 | 5 | T | Ht11(1), Ht13(3) | | | 8 | M1(4), M12(1), M36(1), M46(1), M52(1) | \ | \ |
| 13L | Olevano Romano | 41,861 | 13,038 | 1 | T | Ht13(1) | | | 2 | M1(2) | \ | \ |
| 15L | P. della Mola | 42,156 | 12,149 | 1 | T | Ht6(1) | | | 2 | M1(2) | 2 | F34(2) |
| 16L | Maccarese | 41,891 | 12,276 | 1 | T | Ht1(1) | | | 2 | M1(1), M2(1) | 2 | F60(1), F61(1) |
| 18L | M. della Tolfa | 42,137 | 11,971 | 1 | T | Ht7(1) | | | \ | \ | \ | \ |
| 19L | Foce Verde | 41,387 | 12,924 | 1 | T | Ht1(1) | | | 2 | M1(1), M51(1) | 2 | F62(1), F65(1) |
| 1A | Atri | 42,580 | 13,979 | 1 | A2 | Ha1(1) | | | 2 | M7(1), M54(1) | \ | \ |
| 1U | E45 km117 | 43,319 | 12,386 | 1 | T | Ht10(1) | | | 2 | M1(2) | \ | \ |
| 2U | E45 km93 | 42,989 | 12,375 | 1 | T | Ht11(1) | | | 2 | M1(2) | \ | \ |
| 1T | Giannella | 42,461 | 11,183 | 5 | T | Ht21(1), Ht22(1), Ht23(2), Ht24(2) | | | 10 | M1(4), M2(1), M5(1), M17(1), M33(1), M48(1), M61(1) | 4 | F36(1), F60(3) |
| 2T | Feniglia | 42,418 | 11,239 | 2 | T | Ht17(1), Ht25(1) | | | 4 | M1(1), M9(1), M17(1), M61(1) | 4 | F35(2), F60(2) |
| 4T | Pian della Rasa | 44,035 | 11,053 | 2 | T | Ht20(2) | | | 4 | M2(4) | \ | \ |
| 1M | Tavullia | 43,884 | 12,761 | 5 | T | Ha35(2), Ha36(2), Ha37(1) | | | \ | \ | 2 | F33(2) |
| 2M | Mondaino | 43,873 | 12,648 | 1 | T | Ha37(1) | | | \ | \ | \ | \ |
| 3M | M. Conca | 43,872 | 12,498 | 3 | T | Ha31(2), Ha34(1) | | | \ | \ | 4 | F32(2), F44(2) |
| 1E | Mesola | 44,856 | 12,244 | 7 | T | Ha30(5), Ha31(2) | | | 12 | M2(2), M7(6), M16(1), M53(2), M60(1) | \ | \ |
| 1SA | Alghero | 40,567 | 8,322 | \ | S33 | \ | | | 2 | M1(2) | \ | \ |
| 2SA | Budoni | 40,706 | 9,711 | 2 | S3 | Hs100(2) | | | 4 | M1(2), M17(1), M53(1) | \ | \ |
| 1a | Monte Gargano | 41,786 | 15,845 | 3 | A2 | Ha1(1), Ha2(1), Ha4(1) | | | \ | \ | \ | \ |
| 3a* | Cepagatti | 42,376 | 14,086 | 1 | A2 | Ha1(1) | | | \ | \ | \ | \ |
| 7* | Ostuni | 40,710 | 17,592 | 1 | A2 | Ha13(1) | | | \ | \ | \ | \ |
| 11* | Colli euganei | 45,317 | 11,664 | 1 | A2 | Ha33(1) | | | \ | \ | \ | \ |
| 12a* | Gropello Cairoli | 45,173 | 9,017 | 1 | A2 | Ha31(1) | | | \ | \ | \ | \ |
| 16* | Sibari | 39,768 | 16,482 | 1 | A3 | Hcc7(1) | | | \ | \ | \ | \ |
| 17* | Scalea | 39,806 | 15,803 | 1 | A2 | Ha41(1) | | | \ | \ | \ | \ |
| 18* | Torre Orsaia | 40,130 | 15,473 | 1 | A2 | Ha15(1) | | | \ | \ | \ | \ |
| 19* | Vallo della Lucania | 40,245 | 15,284 | 1 | A2 | Ha15(1) | | | \ | \ | \ | \ |
| 20* | Paestum | 40,418 | 14,990 | 1 | A2 | Ha18(1) | | | \ | \ | \ | \ |
| 21* | Ischia | 40,715 | 13,952 | 1 | A2 | Ha25(1) | | | \ | \ | \ | \ |
| 26* | Punta Ala | 42,807 | 10,757 | 1 | T | Ht19(1) | | | \ | \ | \ | \ |
| 27a* | Firenze | 43,766 | 11,327 | 1 | T | Ht18(1) | | | \ | \ | \ | \ |
| 28* | Monasterace | 38,455 | 16,581 | 1 | S2 | Hcl4(1) | | | \ | \ | \ | \ |
| 30* | Le Castella | 38,908 | 17,026 | 1 | S1 | Hcl24(1) | | | \ | \ | \ | \ |
| 31* | Lamezia Terme | 38,947 | 16,339 | 1 | S1 | Hcl23(1) | | | \ | \ | \ | \ |
| 32* | Serra San Bruno | 38,568 | 16,335 | 1 | S1 | Hcl13(2) | | | \ | \ | \ | \ |
| 34* | Agrigento | 37,296 | 13,573 | 1 | S3 | Hs44(1) | | | \ | \ | \ | \ |
| 35* | Reggio Calabria | 38,174 | 15,668 | 1 | S3 | Hs98(1) | | | \ | \ | \ | \ |
| 36* | Calanna | 38,187 | 15,727 | 1 | S3 | Hs98(1) | | | \ | \ | \ | \ |
| 37* | Aspromonte | 38,255 | 16,027 | 1 | S3 | Hs99(1) | | | \ | \ | \ | \ |
| 38a* | Oristano | 39,879 | 8,580 | 1 | S3 | Hs100(1) | | | \ | \ | \ | \ |
| 38b* | N-W Sardegna | 40,697 | 8,354 | 1 | S3 | Hs100(1) | | | \ | \ | \ | \ |
| 27b* | Bastia | 42,691 | 9,443 | 1 | T | Ht18(1) | | | \ | \ | \ | \ |
| 38c* | Bonifacio | 41,385 | 9,171 | 1 | S3 | Hs100(1) | | | \ | \ | \ | \ |
| 1b*II | Crveni Otok | 45,058 | 13,626 | 1 | A2 | Ha2(1) | | | \ | \ | \ | \ |
| 1c*II | Sveti Jurai | 45,150 | 13,593 | 1 | A2 | Ha2(1) | | | \ | \ | \ | \ |
| 1d | Zadar Ciovo | 43,497 | 16,273 | 1 | A2 | Ha2(1) | | | \ | \ | \ | \ |
| 1e* | Gradac | 43,010 | 17,579 | 1 | A2 | Ha2(1) | | | \ | \ | \ | \ |
| 1f* | Morovnik (Olib) | 44,433 | 14,737 | 1 | A2 | Ha2(1) | | | \ | \ | \ | \ |
| 1g* | Pholib | 44,410 | 14,817 | 1 | A2 | Ha2(1) | | | \ | \ | \ | \ |
| 1h* | Lutrosnjak | 44,363 | 14,577 | 1 | A2 | Ha2(1) | | | \ | \ | \ | \ |
| 1i* | Sestrunj | 44,184 | 14,991 | 1 | A2 | Ha2(2) | | | \ | \ | \ | \ |
| 1l* | Veseljuh (Kornat) | 43,760 | 15,352 | 1 | A2 | Ha2(1) | | | \ | \ | \ | \ |
| 3b*II | Porec | 45,222 | 13,600 | 1 | A2 | Ha1(1) | | | \ | \ | \ | \ |
| 3c*II | Galiner | 45,154 | 13,594 | 1 | A2 | Ha1(1) | | | \ | \ | \ | \ |
| 3e* | Rovinj | 45,069 | 13,639 | 1 | A2 | Ha1(1) | | | \ | \ | \ | \ |
| 3f* | Bale | 45,042 | 13,779 | 1 | A2 | Ha1(1) | | | \ | \ | \ | \ |
| 3g* | Silo | 45,127 | 14,652 | 1 | A2 | Ha1(1) | | | \ | \ | \ | \ |
| 3h* | Baska | 44,967 | 14,740 | 1 | A2 | Ha1(1) | | | \ | \ | \ | \ |
| 3i* | Krk | 45,088 | 14,610 | 1 | A2 | Ha1(1) | | | \ | \ | \ | \ |
| 3l* | Rab | 44,785 | 14,758 | 1 | A2 | Ha1(1) | | | \ | \ | \ | \ |
| 3m* | Velika Palagruza | na | na | 1 | A2 | Ha1(1) | | | \ | \ | \ | \ |
| 4a* | Mala Sestrica | 43,852 | 15,209 | 1 | A2 | Ha8(1) | | | \ | \ | \ | \ |
| 4b* | Duzac | 44,258 | 14,731 | 1 | A2 | Ha8(1) | | | \ | \ | \ | \ |
| 4c* | Crnikova | 44,255 | 14,758 | 1 | A2 | Ha8(1) | | | \ | \ | \ | \ |
| 4d* | Veliki Laganj | 44,166 | 14,808 | 1 | A2 | Ha8(1) | | | \ | \ | \ | \ |
| 12b*II | Antonci | na | na | 1 | A2 | Ha31(1) | | | \ | \ | \ | \ |
| 12c*II | Gusti Skolj | na | na | 1 | A2 | Ha31(1) | | | \ | \ | \ | \ |
| 12d*II | Lakal | 45,161 | 13,593 | 1 | A2 | Ha31(1) | | | \ | \ | \ | \ |
| 12e*II | Lunga | na | na | 1 | A2 | Ha31(1) | | | \ | \ | \ | \ |
| 12f*II | Samer | 45,063 | 13,623 | 1 | A2 | Ha31(1) | | | \ | \ | \ | \ |
| 12g*II | Banjol | na | na | 1 | A2 | Ha31(1) | | | \ | \ | \ | \ |
| 12h*II | Veli Piruzi | na | na | 1 | A2 | Ha31(1) | | | \ | \ | \ | \ |
| 12i*II | Sveti Ivan | na | na | 1 | A2 | Ha31(1) | | | \ | \ | \ | \ |
| 12l*II | Sturag | 45,143 | 13,601 | 1 | A2 | Ha31(1) | | | \ | \ | \ | \ |
| 12m*II | Gustinja | 45,015 | 13,682 | 1 | A2 | Ha31(1) | | | \ | \ | \ | \ |
| 6* | Velika Sestrica | 45,034 | 13,675 | 1 | A2 | Ha7(1) | | | \ | \ | \ | \ |
| 3d* | Vrsar | 45,146 | 13,611 | 3 | A2 | Ha1(1), Ha14(1), Ha31(1) | | | \ | \ | \ | \ |
| 9* | Poljica | 45,092 | 14,484 | 1 | A2 | Ha3(1) | | | \ | \ | \ | \ |
| 13* | Tovarjez | 45,169 | 13,582 | 1 | A2 | Ha32(1) | | | \ | \ | \ | \ |
| 14* | Molat | 44,216 | 14,868 | 1 | A2 | Ha39(1) | | | \ | \ | \ | \ |
| 15* | Ponikva | 45,065 | 14,569 | 1 | A2 | Ha38(1) | | | \ | \ | \ | \ |
| 22* | Susac | 42,757 | 16,514 | 1 | A1 | Hcr3(1) | | | \ | \ | \ | \ |
| 23a* | Pod Kopiste | 42,754 | 16,718 | 1 | A1 | Hcr1(1) | | | \ | \ | \ | \ |
| 23b* | Pijavica | 43,438 | 16,157 | 1 | A1 | Hcr1(1) | | | \ | \ | \ | \ |
| 24* | Mala Palagruza | 42,390 | 16,273 | 1 | A1 | Hcr4(1) | | | \ | \ | \ | \ |
| 25* | Kluda | 43,449 | 16,086 | 1 | A1 | Hcr2(1) | | | \ | \ | \ | \ |
| 29a* | Dubrovnik | 42,679 | 18,066 | 1 | S1 | Hcl18(1) | | | \ | \ | \ | \ |
| 29b* | Kotor | 42,431 | 18,744 | 2 | A2-S1 | Ha8(1), Hcl18(1) | | | \ | \ | \ | \ |
| 39* | Menorca | 39,915 | 4,079 | 1 | S3 | Hs101(1) | | | \ | \ | \ | \ |

Table S1. Sample size of each locality (with relative locality codes). MtDNA and nuDNA haplotypes are also reported for each sampling location. Symbol * indicate samples retived from Podnar et al. 2005; (II) indicate samples which come from Istrian Islands.
