# Supplementary material for: A combination of long term fragmentation and glacial persistence drove the evolutionary history of the Italian wall lizard Podarcis siculus
Source: BMC Evol Biol. 2017 Jan 5;17:6. doi: 10.1186/s12862-016-0847-1 (PMC5216540; doi:10.1186/s12862-016-0847-1)
Supplement: Additional file 2: Table S2. — Voucher with relative accesison numbers and haplotypes. (DOCX 58 kb) [file 12862_2016_847_MOESM2_ESM.docx]

| **voucher** | **Accession number** | ***Cytb* haplotype** | **Accession number** | ***mc1r***  **haplotype(s)** | **Accession number** | ***β, fibint7* haplotype(s)** |
| --- | --- | --- | --- | --- | --- | --- |
| 1aS_273 | KY064841 | Hs1 | KY064579 | M1(2) | / | / |
| 1aS_274 | KY064842 | Hs2 | KY064580 | M1(1), M2(1) | / | / |
| 1aS_275 | / | / | KY064581 | M1(1), M2(1) | / | / |
| 1aS_277 | / | / | KY064582 | M1(2) | / | / |
| 1bS_278 | KY064843 | Hs3 | KY064583 | M2(1), M3(1) | / | / |
| 1bS_279 | KY064844 | Hs2 | KY064584 | M1(1), M4(1) | / | / |
| 1bS_280 | / | / | KY064585 | M1(1), M4(1) | / | / |
| 1bS_281 | KY064845 | Hs1 | KY064586 | M1(1), M2(1) | / | / |
| 1bS_282 | KY064846 | Hs4 | KY064587 | M1(1), M5(1) | / | / |
| 2aS_283 | KY064847 | Hs5 | KY064588 | M1(1), M4(1) | / | / |
| 2aS_284 | KY064848 | Hs6 | KY064589 | M6(1), M7(1) | / | / |
| 2aS_285 | KY064849 | Hs7 | KY064590 | M1(1), M2(1) | / | / |
| 2bS_294 | KY064850 | Hs8 | KY064591 | M1(2) | / | / |
| 3S_ 286 | KY064851 | Hs6 | KY064592 | M1(2) | / | / |
| 3S_ 287 | KY064852 | Hs9 | KY064593 | M1(2) | / | / |
| 4S_ 288 | KY064853 | Hs10 | KY064594 | M1(2) | / | / |
| 4S_ 289 | KY064854 | Hs10 | / | / | / | / |
| 4S_ 290 | KY064855 | Hs6 | KY064595 | M1(1), M8(1) | / | / |
| 4S_ 291 | KY064856 | Hs11 | KY064596 | M2(1), M4(1) | / | / |
| 4S_ 292 | KY064857 | Hs12 | KY064597 | M1(2) | / | / |
| 4S_ 293 | KY064858 | Hs7 | KY064598 | M2(1), M9(1) | / | / |
| 5S_ 271 | KY064859 | Hs13 | KY064599 | M2(1), M10(1) | / | / |
| 6S_ 15 | KY064860 | Hs13 | / | / | KY094514 | F1(1), F2(1) |
| 6S_ 16 | KY064861 | Hs13 | / | / | KY094515 | F1(2) |
| 6S_ 266 | KY064862 | Hs14 | KY064600 | M2(1), M5(1) | / | / |
| 6S_ 267 | KY064863 | Hs15 | KY064601 | M1(2) | / | / |
| 7S_ 262 | KY064864 | Hs13 | KY064602 | M1(2) | / | / |
| 8S_ 258 | KY064865 | Hs16 | KY064603 | M1(2) | / | / |
| 8S_ 259 | KY064866 | Hs17 | KY064604 | M1(1), M4(1) | / | / |
| 8S_ 260 | KY064867 | Hs18 | KY064605 | M1(2) | / | / |
| 8S_ 261 | KY064868 | Hs19 | KY064606 | M1(2) | / | / |
| 9S_ 243 | KY064869 | Hs20 | KY064607 | M1(1), M11(1) | / | / |
| 9S_ 244 | KY064870 | Hs20 | KY064608 | M2(1), M11(1) | / | / |
| 10S_252 | KY064871 | Hs21 | KY064609 | M1(1), M4(1) | / | / |
| 11S_254 | / | / | KY064610 | M1(1), M2(1) | / | / |
| 12S_257 | KY064872 | Hs22 | KY064611 | M1(2) | / | / |
| 13S_94 | KY064873 | Hs25 | KY064612 | M1(2) | / | / |
| 13S_95 | KY064874 | Hs23 | KY064613 | M1(2) | / | / |
| 13S_95b | KY064875 | Hs24 | / | / | / | / |
| 14S_18 | KY064876 | Hs26 | KY064614 | M1(1), M2(1) | / | / |
| 14S_22 | KY064877 | Hs27 | KY064615 | M1(2) | KY094516 | F1(2) |
| 14S_25 | KY064878 | Hs28 | KY064616 | M1(2) | / | / |
| 14S_38 | / | / | KY064617 | M12(2) | / | / |
| 14S_48 | KY064879 | Hs26 | KY064618 | M1(2) | KY094517 | F1(2) |
| 14S_49 | KY064880 | Hs26 | KY064619 | M1(2) | KY094518 | F1(2) |
| 14S_51 | KY064881 | Hs26 | KY064620 | M1(2) | KY094519 | F1(2) |
| 15S_54 | KY064882 | Hs26 | KY064621 | M1(1), M13(1) | / | / |
| 16S_56 | / | / | KY064622 | M1(2) | / | / |
| 16S_57 | KY064883 | Hs29 | KY064623 | M1(2) | KY094520 | F4(2) |
| 16S_59 | KY064884 | Hs30 | KY064624 | M1(1), M2(1) | / | / |
| 16S_61 | KY064885 | Hs31 | KY064625 | M1(2) | KY094521 | F1(1), F4(1) |
| 16S_91 | KY064886 | Hs32 | KY064626 | M1(2) | / | / |
| 16S_93 | KY064887 | Hs30 | KY064627 | M1(2) | / | / |
| 17S_71 | KY064888 | Hs33 | KY064628 | M1(2) | KY094522 | F1(2) |
| 17S_72 | KY064889 | Hs33 | KY064629 | M1(2) | KY094523 | F1(2) |
| 18S_96 | KY064890 | Hs34 | KY064630 | M1(2) | / | / |
| 18S_97 | KY064891 | Hs35 | KY064631 | M1(2) | / | / |
| 18S_98 | KY064892 | Hs35 | KY064632 | M1(2) | / | / |
| 18S_99 | KY064893 | Hs36 | KY064633 | M1(2) | / | / |
| 19S_101 | KY064894 | Hs37 | KY064634 | M1(2) | / | / |
| 19S_102 | KY064895 | Hs37 | KY064635 | M1(2) | / | / |
| 19S_103 | KY064896 | Hs35 | KY064636 | M1(2) | KY094524 | F3(2) |
| 19S_104 | KY064897 | Hs38 | KY064637 | M1(2) | / | / |
| 19S_105 | KY064898 | Hs38 | KY064638 | M1(2) | / | / |
| 20S_128 | KY064899 | Hs39 | KY064639 | M1(2) | / | / |
| 20S_129 | KY064900 | Hs40 | KY064640 | M1(2) | / | / |
| 20S_130 | KY064901 | Hs25 | KY064641 | M1(2) | / | / |
| 21S_136 | KY064902 | Hs41 | KY064642 | M1(1), M14(1) | / | / |
| 21S_137 | KY064903 | Hs39 | KY064643 | M1(2) | / | / |
| 21S_138 | / | / | KY064644 | M1(2) | / | / |
| 22S_141 | KY064904 | Hs42 | KY064645 | M1(2) | / | / |
| 22S_142 | KY064905 | Hs38 | KY064646 | M1(2) | KY094525 | F1(2) |
| 23S_152 | KY064906 | Hs43 | KY064647 | M1(2) | KY094526 | F1(1), F4(1) |
| 24S_26 | KY064907 | Hs38 | KY064648 | M1(1), M15(1) | KY094527 | F1(2) |
| 25S_153 | KY064908 | Hs44 | KY064649 | M1(2) | / | / |
| 25S_154 | KY064909 | Hs45 | / | / | / | / |
| 25S_157 | KY064910 | Hs46 | KY064650 | M1(2) | / | / |
| 25S_157b | KY064911 | Hs45 | KY064651 | M1(2) | / | / |
| 26S_239 | KY064912 | Hs47 | KY064652 | M1(2) | / | / |
| 26S_240 | KY064913 | Hs48 | KY064653 | M7(1), M16(1) | / | / |
| 26S_241 | KY064914 | Hs49 | KY064654 | M1(2) | / | / |
| 26S_242 | KY064915 | Hs50 | KY064655 | M1(2) | / | / |
| 27S_235 | KY064916 | Hs51 | KY064656 | M1(2) | / | / |
| 28S_232 | KY064917 | Hs52 | KY064657 | M1(2) | / | / |
| 28S_233 | KY064918 | Hs53 | KY064658 | M1(2) | / | / |
| 28S_234 | KY064919 | Hs54 | KY064659 | M7(1), M15(1) | / | / |
| 29S_231 | KY064920 | Hs55 | KY064660 | M1(2) | / | / |
| 30S_160 | KY064921 | Hs56 | KY064661 | M1(2) | / | / |
| 30S_161 | KY064922 | Hs56 | / | / | / | / |
| 30S_163 | KY064923 | Hs56 | / | / | / | / |
| 31S_223 | KY064924 | Hs57 | KY064662 | M9(1), M17(1) | / | / |
| 31S_226 | KY064925 | Hs58 | KY064663 | M1(2) | / | / |
| 31S_227 | / | / | KY064664 | M1(1), M2(1) | / | / |
| 31S_228 | / | / | KY064665 | M1(2) | / | / |
| 31S_229 | KY064926 | Hs59 | KY064666 | M1(2) | / | / |
| 31S_230 | / | / | KY064667 | M1(1), M4(1) | / | / |
| 32S_220 | KY064927 | Hs60 | KY064668 | M1(1), M2(1) | / | / |
| 32S_222 | / | / | / | / | / | / |
| 33S_217 | / | / | KY064669 | M4(1), M18(1) | / | / |
| 33S_218 | / | / | KY064670 | M19(2) | / | / |
| 34S_214 | KY064928 | Hs23 | KY064671 | M1(2) | / | / |
| 34S_215 | KY064929 | Hs61 | KY064672 | M1(2) | / | / |
| 35S_29 | KY064930 | Hs40 | KY064673 | M1(2) | KY094528 | F1(2) |
| 36S_30 | KY064931 | Hs62 | KY064674 | M1(1), M20(1) | / | / |
| 36S_31 | KY064932 | Hs63 | KY064675 | M9(1), M17(1) | / | / |
| 36S_32 | KY064933 | Hs64 | KY064676 | M1(2) | KY094529 | F1(2) |
| 36S_34 | KY064934 | Hs63 | KY064677 | M1(2) | / | / |
| 36S_35 | KY064935 | Hs65 | KY064678 | M4(1), M7(1) | / | / |
| 36S_36 | / | / | KY064679 | M1(1), M12(1) | KY094530 | F1(1), F7(1) |
| 36S_37 | KY064936 | Hs66 | KY064680 | M1(2) | / | / |
| 36S_38 | KY064937 | Hs67 | KY064681 | M12(1) | KY094531 | F1(2) |
| 36S_39 | KY064938 | Hs68 | KY064682 | M1(1), M21(1) | KY094532 | F1(2) |
| 36S_40 | KY064939 | Hs23 | KY064683 | M1(2) | KY094533 | F1(2) |
| 36S_41 | KY064940 | Hs23 | / | / | / | / |
| 37S_210 | KY064941 | Hs69 | KY064684 | M1(1), M2(1) | / | / |
| 37S_211 | KY064942 | Hs70 | KY064685 | M2(1), M6(1) | / | / |
| 37S_212 | KY064943 | Hs69 | KY064686 | M1(2) | / | / |
| 38S_209 | KY064944 | Hs71 | KY064687 | M1(1), M2(1) | / | / |
| 39S_165 | KY064945 | Hs72 | KY064688 | M1(1), M12(1) | KY094534 | F4(1), F6(1) |
| 39S_166 | KY064946 | Hs72 | KY064689 | M1(2) | / | / |
| 40S_204 | KY064947 | Hs23 | KY064690 | M1(1), M6(1) | / | / |
| 40S_205 | KY064948 | Hs23 | KY064691 | M1(1), M5(1) | / | / |
| 40S_208 | KY064949 | Hs72 | KY064692 | M1(2) | / | / |
| 41S_203 | KY064950 | Hs73 | KY064693 | M1(2) | / | / |
| 42S_42 | KY064951 | Hs74 | KY064694 | M1(2) | KY094535 | F1(1), F5(1) |
| 42S_44 | KY064952 | Hs75 | KY064695 | M1(2) | KY094536 | F1(2) |
| 43S_199 | KY064953 | Hs75 | KY064696 | M1(2) | / | / |
| 44S_169 | KY064954 | Hs76 | KY064697 | M1(1), M12(1) | KY094537 | F3(2) |
| 44S_170 | KY064955 | Hs77 | KY064698 | M1(2) | / | / |
| 45S_196 | KY064956 | Hs78 | KY064699 | M1(2) | / | / |
| 46S_197 | KY064957 | Hs79 | KY064700 | M1(2) | / | / |
| 47S_182 | KY064958 | Hs80 | KY064701 | M1(1), M2(1) | / | / |
| 47S_183 | KY064959 | Hs80 | KY064702 | M1(2) | / | / |
| 47S_184 | KY064960 | Hs81 | KY064703 | M1(2) | / | / |
| 47S_185 | KY064961 | Hs82 | KY064704 | M1(2) | / | / |
| 47S_187 | / | / | KY064705 | M1(1), M6(1) | / | / |
| 47S_189 | KY064962 | Hs83 | / | / | / | / |
| 48S_190 | KY064963 | Hs84 | KY064706 | M6(1), M22(1) | / | / |
| 48S_191 | KY064964 | Hs85 | KY064707 | M1(2) | / | / |
| 48S_193 | KY064965 | Hs86 | KY064708 | M1(2) | / | / |
| 49S_194 | KY064966 | Hs87 | / | / | / | / |
| 50S_181 | KY064967 | Hs88 | KY064709 | M1(1), M2(1) | / | / |
| 51S_175 | KY064968 | Hs89 | KY064710 | M1(1), M6(1) | / | / |
| 51S_176 | KY064969 | Hs90 | KY064711 | M1(1), M2(1) | / | / |
| 51S_177 | KY064970 | Hs81 | KY064712 | M1(1), M2(1) | / | / |
| 51S_178 | / | / | KY064713 | M1(1), M23(1) | / | / |
| 51S_179 | KY064971 | Hs81 | KY064714 | M1(1), M2(1) | / | / |
| 51S_180 | KY064972 | Hs91 | KY064715 | M1(2) | / | / |
| 52S_01 | / | / | KY064716 | M1(1), M6(1) | / | / |
| 52S_06 | KY064973 | Hs92 | KY064717 | M1(2) | KY094538 | F1(1), F3(1) |
| 52S_07 | KY064974 | Hs93 | KY064718 | M1(1), M24(1) | / | / |
| 53S_02 | KY064975 | Hs94 | KY064719 | M1(2) | KY094539 | F3(2) |
| 53S_03 | KY064976 | Hs94 | KY064720 | M1(2) | KY094540 | F1(2) |
| 53S_04 | KY064977 | Hs95 | KY064721 | M1(2) | KY094541 | F3(2) |
| 53S_05 | KY064978 | Hs94 | KY064722 | M2(1), M15(1) | KY094542 | F1(2) |
| 54S_08 | KY064979 | Hs92 | KY064723 | M1(1), M2(1) | / | / |
| 55S_09 | KY064980 | Hs96 | KY064724 | M1(2) | / | / |
| 55S_11 | KY064981 | Hs81 | KY064725 | M1(2) | KY094543 | F1(1), F3(1) |
| 55S_12 | / | / | KY064726 | M1(2) | / | / |
| 55S_13 | KY064982 | Hs97 | KY064727 | M1(2) | KY094544 | F1(1), F4(1) |
| 1CL_138 | KY064983 | Hcl5 | KY064731 | M25(1), M26(1) | KY094545 | F8(1), F31(1) |
| 1CL_140 | KY064984 | Hcl1 | KY064732 | M1(1), M26(1) | KY094547 | F22(1), F23(1) |
| 2CL_141 | KY064985 | Hcl2 | KY064733 | M1(1), M26(1) | KY094546 | F1(1), F14(1) |
| 2CL_142 | KY064986 | Hcl3 | KY064734 | M1(1), M27(1) | KY094548 | F31(2) |
| 3CL_127 | KY064987 | Hcl6 | / | / | / | / |
| 4CL_113 | KY064988 | Hcl8 | KY064735 | M28(2) | KY094549 | F16(1), F24(1) |
| 4CL_114 | KY064989 | Hcl10 | KY064736 | M29(1), M30(1) | KY094550 | F30(2) |
| 5CL_136 | KY064990 | Hcl13 | KY064737 | M1(1), M31(1) | KY094551 | F17(1), F20(1) |
| 5CL_137 | KY064991 | Hcl13 | KY064738 | M1(2) | KY094552 | F16(1), F29(1) |
| 6CL_129 | KY064992 | Hcl7 | KY064739 | M1(1), M28(1) | KY094553 | F9(1), F27(1) |
| 7CL_144 | KY064993 | Hcl12 | KY064740 | M28(2) | KY094554 | F16(1), F18(1) |
| 7CL_145 | KY064994 | Hcl9 | / | / | KY094555 | F15(1), F25(1) |
| 7CL_146 | KY064995 | Hcl11 | KY064741 | M28(1), M29(1) | KY094556 | F27(1), F28(1) |
| 8CL_148 | KY064996 | Hcl20 | / | / | KY094557 | F16(2) |
| 8CL_150 | KY064997 | Hcl19 | KY064742 | M28(1), M32(1) | KY094558 | F16(2) |
| 11CL_119 | KY064998 | Hcc3 | / | / | / | / |
| 12CL_151 | KY064999 | Hcc1 | KY064743 | M1(1), M35(1) | KY094559 | F15(1), F26(1) |
| 12CL_152 | KY065000 | Hcc1 | KY064744 | M1(2) | KY094560 | F10(1), F12(1) |
| 12CL_154 | KY065001 | Hcc1 | KY064745 | M32(1), M35(1) | KY094561 | F13(1), F52(1) |
| 13CL_115 | KY065002 | Hcc3 | KY064746 | M28(2) | / | / |
| 13CL_116 | KY065003 | Hcc2 | / | / | / | / |
| 14CL_110 | KY065004 | Hcc4 | KY064748 | M28(1), M35(1) | KY094562 | F27(2) |
| 14CL_111 | KY065005 | Hcc5 | KY064747 | M1(1), M35(1) | KY094563 | F16(2) |
| 14CL_112 | KY065006 | Hcc6 | KY064749 | M28(2) | KY094564 | F16(2) |
| 16CL_101 | KY065007 | Ha40 | KY064750 | M36(2) | / | / |
| 16CL_102 | / | / | KY064751 | M34(2) | / | / |
| 16CL_103 | / | / | KY064752 | M37(1), M38(1) | / | / |
| 17CL_105 | KY065008 | Ha44 | KY064753 | M2(1), M34(1) | / | / |
| 18CL_106 | KY065009 | Ha43 | KY064754 | M33(1), M34(1) | / | / |
| 18CL_107 | KY065010 | Ha42 | KY064755 | M33(1), M34(1) | / | / |
| 20CL_156 | KY065011 | Hcl14 | KY064756 | M1(1), M29(1) | / | / |
| 20CL_157 | KY065012 | Hcl15 | KY064757 | M1(1), M62(1) | / | / |
| 20CL_158 | / | / | KY064758 | M62(1), M65(1) | / | / |
| 20CL_159 | KY065013 | Hcl14 | KY064759 | M29(1), M62(1) | KY094565 | F11(1), F19(1) |
| 20CL_160 | / | / | KY064760 | M1(1), M6(1) | / | / |
| 21CL_164 | KY065014 | Hcl21 | KY064761 | M21(1), M64(1) | / | / |
| 21CL_167 | / | / | KY064762 | M1(1), M62(1) | / | / |
| 22CL_170 | / | / | KY064763 | M62(1), M63(1) | / | / |
| 22CL_171 | KY065015 | Hcl22 | KY064764 | M1(1), M66(1) | KY094566 | F16(1), F18(1) |
| 22CL_172 | KY065016 | Hcl16 | KY064765 | M1(1), M29(1) | / | / |
| 22CL_173 | KY065017 | Hcl17 | KY064766 | M28(2) | KY094567 | F16(2) |
| 1P_ 06 | KY065018 | Ha9 | / | / | KY094568 | F21(2) |
| 2P_ 07 | KY065019 | Ha6 | KY064767 | M2(2) | / | / |
| 3P_ 08 | KY065020 | Ha12 | KY064768 | M2(1), M39(1) | KY094569 | F42(2) |
| 4P_ 09 | KY065021 | Ha1 | KY064769 | M4(1), M40(1) | KY094570 | F43(1), F47(1) |
| 4P_ 10 | KY065022 | Ha1 | KY064770 | M41(1), M42(1) | / | / |
| 4P_ 11 | KY065023 | Ha1 | / | / | KY094571 | F41(1), F46(1) |
| 4P_ 12 | KY065024 | Ha2 | / | / | KY094572 | F50(1), F51(1) |
| 4P_ 13 | KY065025 | Ha1 | KY064771 | M43(1), M44(1) | / | / |
| 4P_ 14 | KY065026 | Ha1 | KY064772 | M2(1), M44(1) | / | / |
| 1C_ 14 | KY065027 | Ha15 | KY064773 | M34(2) | KY094573 | F56(2) |
| 1C_ 15 | KY065028 | Ha17 | KY064774 | M34(2) | KY094574 | F52(2) |
| 1C_ 16 | KY065029 | Ha15 | KY064775 | M34(2) | KY094575 | F57(1), F58(1) |
| 1C_ 17 | KY065030 | Ha15 | KY064776 | M34(2) | / | / |
| 1C_ 18 | KY065031 | Ha15 | / | / | / | / |
| 3C_ 13 | KY065032 | Ha22 | KY064777 | M34(1), M55(1) | / | / |
| 4C_ 07 | / | / | KY064778 | M56(1), M57(1) | / | / |
| 4C_ 08 | KY065033 | Ha20 | / | / | / | / |
| 4C_ 09 | / | / | KY064779 | M1(1), M56(1) | KY094576 | F44(2) |
| 4C_ 10 | KY065034 | Ha19 | / | / | / | / |
| 4C_ 11 | KY065035 | Ha21 | KY064780 | M34(2) | KY094577 | F45(2) |
| 4C_ 12 | KY065036 | Ha15 | KY064781 | M34(1), M42(1) | / | / |
| 5C_ 05 | KY065037 | Ha15 | KY064782 | M2(1), M34(1) | / | / |
| 5C_ 06 | KY065038 | Ha15 | KY064783 | M57(1), M58(1) | / | / |
| 6C_ 02 | KY065039 | Ha15 | KY064784 | M2(1), M34(1) | / | / |
| 6C_ 03 | KY065040 | Ha24 | KY064785 | M34(1), M58(1) | KY094578 | F44(1), F45(1) |
| 7C_ 19 | KY065041 | Ha15 | / | / | KY094579 | F45(2) |
| 7C_ 20 | KY065042 | Ha16 | / | / | KY094580 | F39(1), F45(1) |
| 7C_ 21 | KY065043 | Ha16 | / | / | / | / |
| 7C_ 22 | KY065044 | Ha5 | / | / | / | / |
| 8C_ 23 | KY065045 | Ha23 | / | / | / | / |
| 9C_ 24 | KY065046 | Ha10 | / | / | KY094581 | F37(1), F48(1) |
| 9C_ 25 | KY065047 | Ha10 | / | / | / | / |
| 9C_ 26 | KY065048 | Ha29 | / | / | / | / |
| 9C_ 27 | KY065049 | Ha10 | / | / | / | / |
| 10C_30 | KY065050 | Ha27 | KY064786 | M34(2) | KY094582 | F38(1), F40(1) |
| 10C_31 | KY065051 | Ha27 | KY064787 | M17(1), M34(1) | / | / |
| 10C_32 | KY065052 | Ha28 | KY064788 | M34(2) | KY094583 | F49(2) |
| 11C_33 | KY065053 | Ha26 | KY064789 | M34(2) | / | / |
| 12C_41 | KY065054 | Ha11 | KY064790 | M2(1), M59(1) | KY094584 | F54(1), F55(1) |
| 12C_42 | KY065055 | Ha11 | KY064791 | M6(1), M39(1) | KY094585 | F44(1), F53(1) |
| 1B_ 07 | KY065056 | Ha1 | KY064792 | M2(1), M5(1) | / | / |
| 1L_ 120 | KY065057 | Ht10 | KY064793 | M1(1), M34(1) | / | / |
| 2L_ 46 | KY065058 | Ht1 | KY064794 | M2(1), M45(1 | / | / |
| 2L_ 47 | KY065059 | Ht10 | KY064795 | M1(1), M34(1) | KY094586 | F63(2) |
| 2L_ 48 | KY065060 | Ht13 | KY064796 | M21(1), M46(1 | / | / |
| 3L_ 19 | KY065061 | Ht12 | KY064797 | M1(2) | / | / |
| 4L_ 49 | KY065062 | Ht4 | KY064799 | M1(1), M34(1) | / | / |
| 4L_ 50 | KY065063 | Ht12 | KY064800 | M1(2) | KY094587 | F60(2) |
| 4L_ 51 | KY065064 | Ht1 | KY064801 | M1(2) | KY094588 | F64(1), F66(1) |
| 4L_ 52 | KY065065 | Ht1 | KY064802 | M1(1), M26(1) | / | / |
| 4L_ 53 | KY065066 | Ht12 | KY064803 | M1(1), M26(1) | / | / |
| 4L_ 20 | KY065067 | Ht1 | KY064798 | M1(1), M26(1) | / | / |
| 9L_ 23 | KY065068 | Ht9 | KY064804 | M1(2) | KY094589 | F34(1), F60(1) |
| 9L_ 39 | KY065069 | Ht8 | KY064805 | M1(2) | KY094590 | F62(2) |
| 9L_ 40 | KY065070 | Ht3 | KY064806 | M1(1), M47(1) | / | / |
| 9L_ 41 | KY065071 | Ht1 | KY064807 | M1(1), M48(1) | KY094591 | F68(2) |
| 9L_ 42 | / | / | KY064808 | M1(2) | KY094592 | F59(1), F60(1) |
| 9L_ 43 | KY065072 | Ht5 | KY064809 | M1(1), M48(1) | / | / |
| 9L_ 44 | KY065073 | Ht1 | KY064810 | M1(1), M49(1) | / | / |
| 10L_32 | KY065074 | Ht2 | KY064811 | M15(1), M50(1) | KY094593 | F60(2) |
| 10L_34 | KY065075 | Ht2 | KY064812 | M12(1), M51(1) | KY094594 | F34(2) |
| 11L_71 | KY065076 | Ht13 | KY064813 | M1(2) | / | / |
| 11L_72 | KY065077 | Ht13 | KY064814 | M1(1), M2(1) | / | / |
| 12L_11 | KY065078 | Ht13 | KY064815 | M1(2) | / | / |
| 12L_12 | KY065079 | Ht14 | / | / | / | / |
| 12L_13 | KY065080 | Ht11 | KY064816 | M1(1), M52(1) | / | / |
| 12L_14 | KY065081 | Ht13 | KY064817 | M1(1), M46(1) | / | / |
| 12L_74 | KY065082 | Ht13 | KY064818 | M12(1), M36(1) | / | / |
| 13L_75 | KY065083 | Ht13 | KY064819 | M1(2) | / | / |
| 15L_30 | KY065084 | Ht6 | KY064820 | M1(2) | KY094595 | F34(2) |
| 16L_15 | KY065085 | Ht1 | KY064821 | M1(1), M2(1) | KY094596 | F60(1), F61(1) |
| 18L_05 | KY065086 | Ht7 | / | / | / | / |
| 19L_18 | KY065087 | Ht1 | KY064822 | M1(1), M51(1) | KY094597 | F62(1), F65(1) |
| 1A_ 01 | KY065088 | Ha1 | KY064823 | M7(1), M54(1) | / | / |
| 1U_ 01 | KY065089 | Ha16 | KY064824 | M1(2) | / | / |
| 2U_ 02 | KY065090 | Ht15 | KY064825 | M1(2) | / | / |
| 1T_ 03 | KY065091 | Ht22 | KY064826 | M1(2) | KY094598 | F36(1), F60(1) |
| 1T_ 04 | KY065092 | Ht23 | KY064827 | M1(1), M61(1) | / | / |
| 1T_ 05 | KY065093 | Ht24 | KY064828 | M1(1), M48(1) | KY094599 | F60(2) |
| 1T_ 06 | KY065094 | Ht24 | KY064829 | M5(1), M33(1) | / | / |
| 1T_ 09 | KY065095 | Ht21 | KY064830 | M2(1), M17(1) | / | / |
| 2T_ 07 | KY065096 | Ht25 | KY064831 | M1(1), M61(1) | KY094600 | F60(2) |
| 2T_ 08 | KY065097 | Ht16 | KY064832 | M9(1), M17(1) | KY094601 | F35(2) |
| 4T_ 10 | KY065098 | Ht20 | KY064833 | M2(2) | / | / |
| 4T_ 11 | KY065099 | Ht20 | KY064834 | M2(2) | / | / |
| 1M_ 07 | KY065100 | Ha35 | / | / | / | / |
| 1M_ 08 | KY065101 | Ha35 | / | / | / | / |
| 1M_ 09 | KY065102 | Ha36 | / | / | KY094602 | F33(2) |
| 1M_ 10 | KY065103 | Ha37 | / | / | / | / |
| 1M_ 11 | KY065104 | Ha36 | / | / | / | / |
| 2M_ 12 | KY065105 | Ha37 | / | / | / | / |
| 3M_ 15 | KY065106 | Ha34 | / | / | KY094603 | F44(2) |
| 3M_ 16 | KY065107 | Ha31 | / | / | KY094604 | F32(2) |
| 3M_ 17 | KY065108 | Ha31 | / | / | / | / |
| 1E_ 01 | KY065109 | Ha30 | KY064835 | M2(1), M7(1) | / | / |
| 1E_ 02 | KY065110 | Ha30 | KY064836 | M7(1), M53(1) | / | / |
| 1E_ 03 | KY065111 | Ha30 | KY064837 | M7(1), M60(1) | / | / |
| 1E_ 04 | KY065112 | Ha30 | KY064838 | M7(1), M53(1) | / | / |
| 1E_ 05 | KY065113 | Ha31 | KY064839 | M2(1), M16(1) | / | / |
| 1E_ 06 | KY065114 | Ha31 | KY064840 | M7(2) | / | / |
| 1E_ 08 | KY065115 | Ha30 | / | / | / | / |
| 1SA_04 | / | / | KY064728 | M17(1), M53(1) | / | / |
| 1SA_10 | KY065116 | Hs100 | KY064729 | M2(2) | / | / |
| 1SA_11 | KY065117 | Hs100 | KY064730 | M2(2) | / | / |
|  |  |  |  |  |  |  |
|  |  |  |  |  |  |  |
|  |  |  |  |  |  |  |
|  |  |  |  |  |  |  |
|  |  |  |  |  |  |  |
|  |  |  |  |  |  |  |
|  |  |  |  |  |  |  |
|  |  |  |  |  |  |  |
|  |  |  |  |  |  |  |
|  |  |  |  |  |  |  |
|  |  |  |  |  |  |  |
|  |  |  |  |  |  |  |
|  |  |  |  |  |  |  |
|  |  |  |  |  |  |  |
|  |  |  |  |  |  |  |
|  |  |  |  |  |  |  |
|  |  |  |  |  |  |  |
|  |  |  |  |  |  |  |
|  |  |  |  |  |  |  |
|  |  |  |  |  |  |  |
