# Supplementary figures and images for: A combination of long term fragmentation and glacial persistence drove the evolutionary history of the Italian wall lizard Podarcis siculus
Source: BMC Evol Biol. 2017 Jan 5;17:6. doi: 10.1186/s12862-016-0847-1 (PMC5216540; doi:10.1186/s12862-016-0847-1)

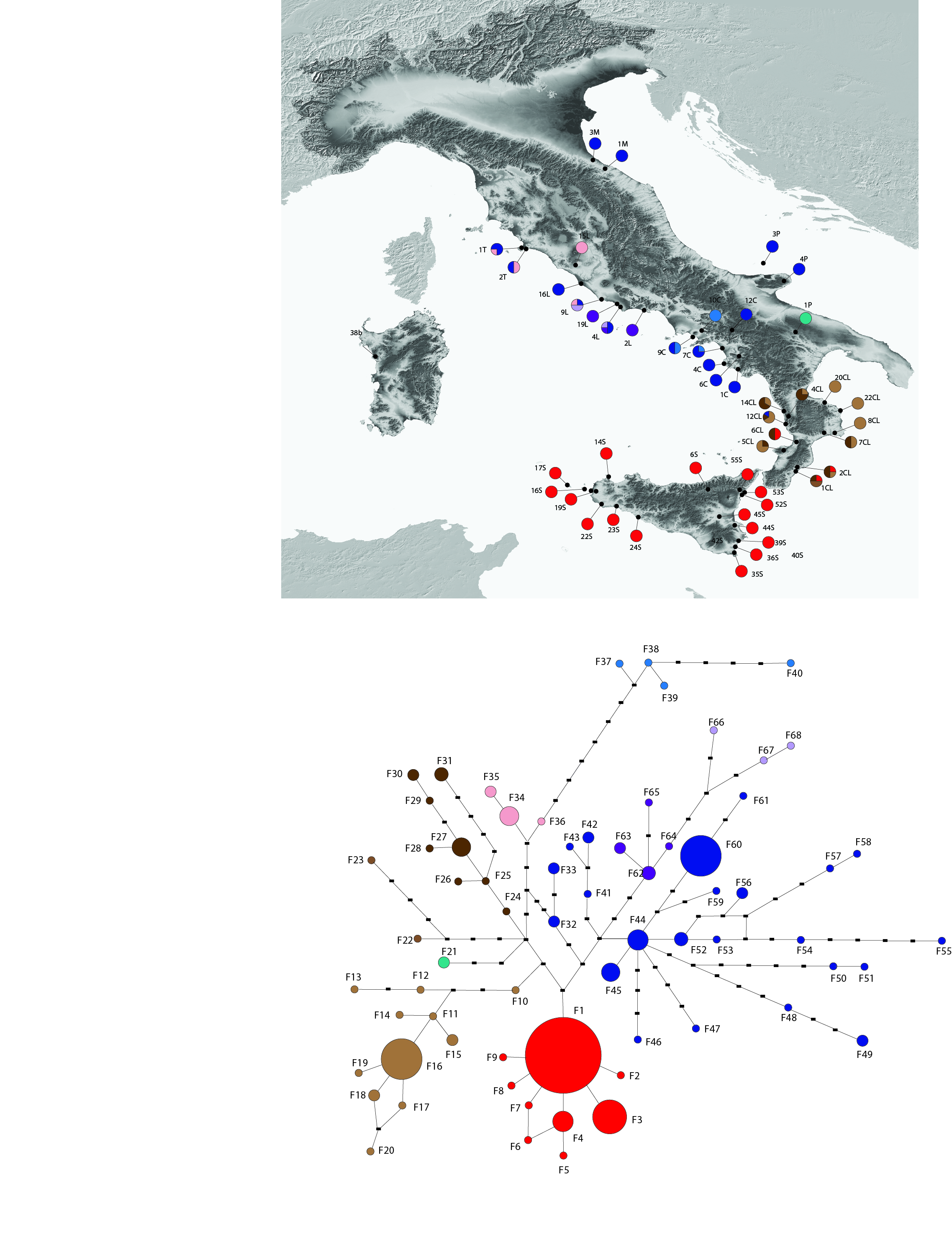

Supplement: Additional file 3: Figure S3. — Geographic allele distribution of β-fibint7 with relative frequencies indicated by pie diagrams at each sampled location. (JPG 4081 kb) [file 12862_2016_847_MOESM3_ESM.jpg]

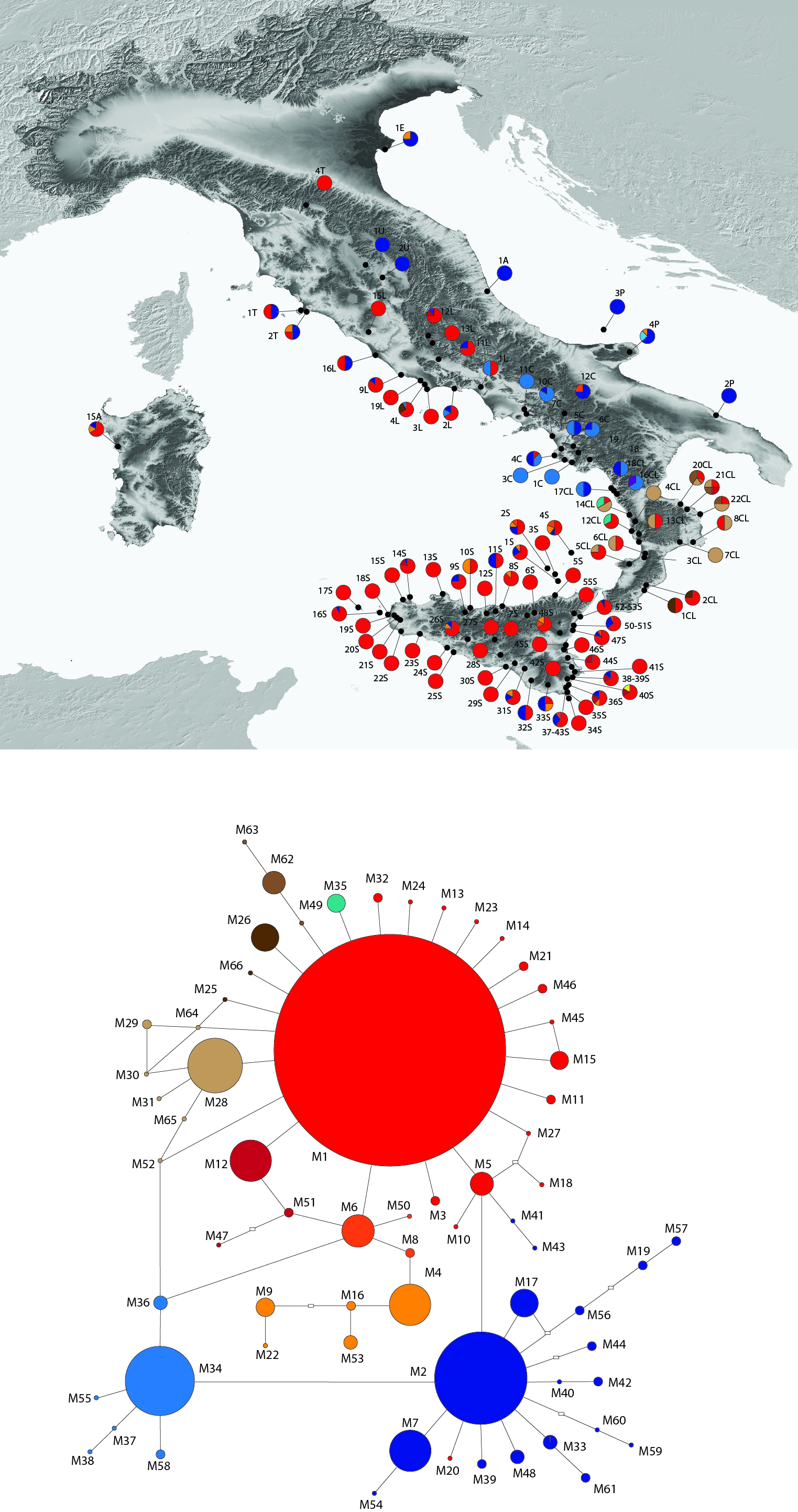

Supplement: Additional file 4: Figure S4. — Geographic allele distribution of mc1r with relative frequencies indicated by pie diagrams at each sampled location. (JPG 4161 kb) [file 12862_2016_847_MOESM4_ESM.jpg]
